# Splenic Nerve Neuromodulation Reduces Inflammation and Promotes Resolution in Chronically Implanted Pigs

**DOI:** 10.3389/fimmu.2021.649786

**Published:** 2021-03-29

**Authors:** David M. Sokal, Alex McSloy, Matteo Donegà, Joseph Kirk, Romain A. Colas, Nikola Dolezalova, Esteban A. Gomez, Isha Gupta, Cathrine T. Fjordbakk, Sebastien Ouchouche, Paul B. Matteucci, Kristina Schlegel, Rizwan Bashirullah, Dirk Werling, Kim Harman, Alison Rowles, Refet Firat Yazicioglu, Jesmond Dalli, Daniel J. Chew, Justin D. Perkins

**Affiliations:** ^1^ Translation and Engineering, Galvani Bioelectronics, Stevenage, United Kingdom; ^2^ Clinical Science & Services, The Royal Veterinary College, Hatfield, United Kingdom; ^3^ Centre for Inflammation and Therapeutic Innovation, Queen Mary University of London, London, United Kingdom; ^4^ Department of Surgery, University of Cambridge, Cambridge, United Kingdom; ^5^ Department of Pathobiology and Population Sciences, Royal Veterinary College, Hatfield, United Kingdom; ^6^ Non-Clinical Safety, GlaxoSmithKline, Ware, United Kingdom

**Keywords:** autonomic nervous system, neuromodulation, stimulation, endotoxemia, specialized pro resolving mediators, inflammation, bioelectronic medicine, splenic nerve

## Abstract

Neuromodulation of the immune system has been proposed as a novel therapeutic strategy for the treatment of inflammatory conditions. We recently demonstrated that stimulation of near-organ autonomic nerves to the spleen can be harnessed to modulate the inflammatory response in an anesthetized pig model. The development of neuromodulation therapy for the clinic requires chronic efficacy and safety testing in a large animal model. This manuscript describes the effects of longitudinal conscious splenic nerve neuromodulation in chronically-implanted pigs. Firstly, clinically-relevant stimulation parameters were refined to efficiently activate the splenic nerve while reducing changes in cardiovascular parameters. Subsequently, pigs were implanted with a circumferential cuff electrode around the splenic neurovascular bundle connected to an implantable pulse generator, using a minimally-invasive laparoscopic procedure. Tolerability of stimulation was demonstrated in freely-behaving pigs using the refined stimulation parameters. Longitudinal stimulation significantly reduced circulating tumor necrosis factor alpha levels induced by systemic endotoxemia. This effect was accompanied by reduced peripheral monocytopenia as well as a lower systemic accumulation of CD16^+^CD14^high^ pro-inflammatory monocytes. Further, lipid mediator profiling analysis demonstrated an increased concentration of specialized pro-resolving mediators in peripheral plasma of stimulated animals, with a concomitant reduction of pro-inflammatory eicosanoids including prostaglandins. Terminal electrophysiological and physiological measurements and histopathological assessment demonstrated integrity of the splenic nerves up to 70 days post implantation. These chronic translational experiments demonstrate that daily splenic nerve neuromodulation, *via* implanted electronics and clinically-relevant stimulation parameters, is well tolerated and is able to prime the immune system toward a less inflammatory, pro-resolving phenotype.

## Introduction

Extensive evidence exists demonstrating that the efferent arm of the “inflammatory reflex” controls systemic immune responses *via* neural circuits that target lymphoid organs, in particular the spleen (1–3). The spleen is innervated by the splenic nerve (SpN), which consists of an abundant network of interconnecting fibers originating from abdominal ganglia (4, 5). This neuronal plexus runs along the splenic artery (SpA), together forming a neurovascular bundle (NVB), until it enters the splenic parenchyma where it releases neurotransmitters, in particular catecholamines, which subsequently modulate immune cells. The spleen plays a major role as a reservoir of monocytes/macrophages and lymphocytes. These cells are activated during infection and inflammation, resulting in the production of cytokines and chemokines and in subsequent mobilization toward sites of inflammation/damage (6, 7).

Exogenous electrical activation of neural pathways targeting the spleen, either *via* upstream activation of the cervical vagus nerve (VN), or near end-organ activation of the SpN, has been shown to induce cytokine modulation in small animals (2, 8–10). Using acute inflammatory models, this immunomodulatory effect has typically been demonstrated as a reduction in cytokine responses, particularly tumor necrosis factor alpha (TNF-α) (2, 8, 11). This effect has been shown to be dependent on the SpN, and on noradrenaline (NA) released from nerve terminals onto splenic leukocytes (8, 11). In addition, electrical stimulation of such neural pathways has been shown to reduce inflammation in animal models of arthritis (10, 12, 13) and inflammatory bowel disease (IBD) (14, 15). Importantly, clinical evidence from feasibility studies of cervical VN stimulation suggests that neuromodulation of these pathways could be beneficial in patients with rheumatoid arthritis (RA) (16, 17) and Crohn’s disease (18). Work from our group and others has provided evidence that these mechanisms also exist in the pig (4), and other large animals (15, 19) with gross anatomy, histology, physiology and immune functions more closely associated to humans. When compared to small animals, the use of large animal models is more appropriate to answer questions on human-relevant clinical device design and therapy translation.

Neuromodulation of the immune system targeting the splenic circuits represents a new avenue for future treatment of patients affected by acute and chronic inflammatory conditions. The SpN represents a suitable target for such an approach, due to its fundamental role in regulating immunological responses as well as its proximity to the target organ, allowing direct splenic activation while potentially avoiding some off-target effects seen with more indirect distal intervention sites of the autonomic nervous system. We have shown that the pig represents a suitable pre-clinical model to investigate and refine neuromodulation therapies targeting the SpN (4). Acute activation of the SpN, using human-relevant stimulation parameters, reduces production of TNF-α and IL-6 following endotoxemia in terminally-anesthetized pigs. In addition, we demonstrated that SpN stimulation (SpNS) caused amplitude- and frequency-dependent changes in SpA blood flow (BF) and systemic mean arterial blood pressure (mABP), which are directly correlated to nerve activation. These acute systemic physiological biomarkers provide a surrogate of target engagement and can be used to determine nerve activation and allow the refinement of neuromodulation parameters. Based on these findings, the feasibility and safety of acute SpN neuromodulation, during minimally-invasive surgery for esophageal cancer (esophagectomy), is currently being evaluated to assess impact on inflammatory and physiological responses (clinicaltrials.gov Identifier: NCT04171011).

The translation from an acute model to a potential chronic therapeutic modality however requires further elucidation. Given the differences between small rodent and human models, conscious large-animal studies investigating the effects of long-term nerve stimulation are a fundamental step toward clinical translation (20). They are needed to refine tolerable and efficacious stimulation parameters, provide evaluation of safety and long-term integration of the system, and develop understanding of long-term neuromodulation on physiological functions. This is particularly crucial for immunomodulatory therapies, since general anesthesia is known to affect immunological functions (21). In addition, large animal models can reveal biological effects previously not studied in rodent models. For example, it has been demonstrated by our group and others, that SpN or VN neuromodulation promotes cardiovascular protection in endotoxemia models in pigs *via* a mechanism independent of cytokine modulation (4, 22), suggestive of regulation of additional pathways beyond cytokine production.

It has been shown that the autonomic nervous system may regulate dynamic mechanisms associated with leukocyte recruitment and resolution processes during inflammation (23, 24), with neurotransmitters and the spleen possibly playing a central role. Resolution of inflammation is an active process requiring fine regulation of biosynthetic pathways that lead to the production of lipid mediators, called specialized pro-resolving mediators (SPMs) (25). SPMs play a central role in reprogramming both innate and adaptive immune responses to regulate immune cell recruitment and cytokine production. Loss of vagal signaling leads to a disruption in SPM biosynthesis and disrupted resolution mechanisms (23, 24). Therefore, it is possible that the same neural circuits that modulate cytokine responses could affect the regulation of SPMs to promote inflammation resolution, potentially broadening the field of investigation for the role of autonomic neuromodulation in inflammatory disorders.

Herein we describe the refinement of stimulation parameters suitable to achieve effective SpN activation and neurotransmitter release, with reduced off-target effects in terminally-anesthetized pigs. Subsequently, pigs were implanted using a novel, minimally-invasive laparoscopic surgical procedure to place a circumferential cuff electrode interface around the splenic NVB, connected to a subcutaneous implantable pulse generator (IPG) to enable the delivery of chronic neuromodulation. Following recovery from surgery, the tolerability of electrical stimulation of the SpN was then evaluated in these conscious, freely-behaving pigs. Subsequently, multiple immunological parameters were quantified in both naïve and endotoxin-challenged inflammatory conditions. Specifically, cytokine production, cell phenotypic changes and SPMs were analyzed in peripheral blood prior to, and after a systemic immune challenge with lipopolysaccharide (LPS), comparing SpN stimulated and non-stimulated “sham” animals. Finally, terminal contrast angiography, electrophysiology and histopathology were performed to evaluate the integrity of the splenic NVB after chronic implantation.

## Materials And Methods

All animal studies were ethically reviewed and carried out in accordance with Animals (Scientific Procedures) Act 1986. The protocol was approved by the Royal Veterinary College Animal Welfare and Ethical Review Board and the Galvani Bioelectronics Animal and Scientific Review Committee. All animals were transported and housed under conditions specified in the United Kingdom Animal Welfare Act 2006 and The Welfare of Farm Animals (England) Regulations 2007.

### Stimulation Parameters

Stimulation parameters and paradigms used are described in the relevant sections in the methods and results and are summarized in **Supplementary Table S5** and **Supplementary Figure S13**.

### Acute Terminal Study Methods

Additional details are described in **Supplementary Methods**.

#### Refinement of SpNS Parameters – Electrophysiology

##### Animals

5 female farm pigs (Large white pigs; body weight 45 – 50 kg, age 10 – 12 weeks) were sourced from a commercial pig farm. On the day of the experiment, anesthesia induction was performed as described for chronic conscious animals below.

##### Surgery

Commencing at the origin of the SpA, a 10 mm long segment of the artery with an intact periarterial SpN plexus was carefully separated from the splenic vein (SpV) and surrounding connective tissue.

##### Electrophysiology

The NVB was subsequently instrumented with a bipolar circumferential cuff electrode (see **Supplementary Methods**).

A distal discrete SpN fascicle was carefully isolated and subsequently instrumented with a bipolar cuff electrode (see **Supplementary Methods**) to record evoked compound action potentials (eCAP). For monitoring blood flow changes during SpNS, an ultrasonic transit time flow probe (Transonics, USA) was placed on the SpA immediately proximal to the electrodes. Electrophysiological and physiological parameters were recorded as detailed in **Supplementary Methods**. The splenic NVB was stimulated *via* the periarterial cuff electrode (described in **Supplementary Table S1**). eCAPs and physiological parameters were subsequently recorded and quantified as described in **Supplementary Methods**.

#### Refinement of SpNS Parameters - Noradrenaline Output

##### Animals

5 female farm pigs (Large white pigs; body weight ~70 kg, age ~5 months) were used; animal sourcing, management and anesthesia protocols were as described above.

##### Surgery

Anesthesia, placement of bilateral jugular venous catheters and a femoral arterial catheter and monitoring and recording of all parameters were performed as described above. Open laparoscopic surgery and isolation of the splenic NVB followed by cuff electrode and flow probe placement was performed as above.

After probe placement, a cannula (12G, 3.25”; Angiocath, BD, UK) was inserted such that its tip lay within the common SpV. The base of the spleen was reintroduced into the abdomen.

##### Stimulation and Blood Sampling

The effects of SpN stimulation on blood flow and NA concentration in the SpV were tested at 6 stimulation intensities, inclusive of sham. Summary of stimulation parameters are shown in **Supplementary Table S5**. The different stimulation intensities were delivered to each animal in a randomized order, using a truncated 6x6 randomly-generated Latin Square design.

Blood was sampled before and during SpNS as described in **Supplementary Methods**. Following each stimulation intensity, a period of 60 mins was allowed for the preparation to re-stabilize before performing the next stimulation. Physiological parameters and blood flows were continuously captured using the Powerlab system (AD Instruments). Following the final stimulation and sampling period, the animal was euthanized by injection of pentobarbital.

##### NA Analysis

Frozen plasma aliquots were analyzed and quantified as described in **Supplementary Methods**.

### Chronic Conscious Neuromodulation Study Methods

The overall study design is outlined in **Figure 3A** and is described in more detail in **Supplementary Methods**.

#### Animals

A total of 12 female Berkshire pigs (74-99 kg at start of study; ~9 months old) were used for the chronic implant study. Female pigs were used due to their temperament and smaller size. The pigs were sourced from a commercial pig farm, acclimatized at the research facility and underwent handling and socialization training for a minimum of 1 month prior to the experiment.

#### Neuromodulation Device

The stimulation lead and implantable pulse generator (IPG) are described in the **Supplementary Methods**.

#### Surgery

##### Implantation of the Neuromodulation Device

Animals were anesthetized and underwent minimally invasive laparoscopic surgery *via* a left sided lateral approach (**Figures 3B, C** **)**. Following retraction of the stomach and then spleen, the splenic NVB was dissected free from the surrounding tissue and the circumferential cuff electrode was implanted around the SpNVB.

Stimulation applied with an external pulse generator (EPG (DS5, Digitimer, UK)) confirmed electrical integrity of the implanted stimulation lead and physiological functionality by a measured increase in systemic mABP. The IPG was implanted in a subcutaneous pocket on the dorso-lateral thorax and the lead tunneled to connect to the IPG before surgical closure of all incisions.

##### Intra-Operative Neuromodulation of the SpN (at Surgery and at Termination)

At implantation, neuromodulation was delivered at either 15 or 40 µC using a 10 Hz continuous paradigm to confirm target engagement (see **Supplementary Methods**). Electrode impedance was measured throughout the course of the study.

At termination, stimulation was delivered using either the 10 Hz continuous or burst paradigm with an EPG up to 40 µC with measurement and assessment of SpA BF and mABP as described above.

##### Vascular Access Port Implantation

All animals were subsequently implanted with an intravenous catheter in the left external jugular vein, using a minimally-invasive ultrasound-guided approach, which was terminated with a subcutaneous vascular access port (**Supplementary Methods**) to enable repeated, stress free, blood sampling in conscious animals.

##### Recovery

Animals were routinely recovered from anesthesia and returned to their pens. Recovery was assessed by return to normal behaviors, pain scoring, wound healing, and body weight.

#### Determination of Effects of Chronic SpN Neuromodulation

##### Tolerability to SpNS as Determined by Behavioral Responses

Animals were given a 14 day recovery period following surgery after which only those assigned to the neuromodulation group (n=6) were stimulated at either 15 or 40 µC using the 10 Hz burst paradigm. Stimulation was tested at ascending 1 mA (2 µC) intervals. For each stimulation current amplitude, stimulation was ramped-up over 30 s and then held for a further 60 s (90 s total).

During neuromodulation, animals were observed by two independent trained assessors for any responses indicative of perception of stimulation (scoring system and observed responses are described in **Supplementary Methods**).

##### Baseline Blood Testing

Animals were then maintained for a further 14 day period without stimulation, after which time all animals underwent baseline blood sampling taken from the VAP for hematology, clinical biochemistry, *ex vivo* LPS cytokine assays and flow cytometry (only performed in cohorts 3 and 4), nominally at day -2, -1 and 0, at the same time of day (around 10:00 h) (see **Figure 3A** for overall study design).

##### Biomarker Assays in Naïve animals

To determine the effects of SpNS in naïve animals, chronic neuromodulation therapy (10 Hz burst at IPG output of either 15 or 40 µC, 7 days a week (see **Supplementary Table S5** for stimulation parameters)) was initiated from Day 0, following the baseline testing. Cohorts 1 and 2 (n=6) received neuromodulation 6 times per day from 09:00 at 90 min intervals, while in cohorts 3 and 4 (n=6), neuromodulation was performed 12 times per day from 07:00 at 60 min intervals. Animals assigned to the sham group (n=6) were not stimulated.

On Days 2 and 7, scheduled neuromodulation was stopped after the 09:00 stimulation and blood drawn from all animals around 10:00 for *ex vivo* LPS cytokine assays (plus, additional samples per baseline bloods as described above). Scheduled neuromodulation was then restarted so the animals received their 11:00 therapy session.

###### Ex vivo LPS Cytokine Assays

Duplicate samples of peripheral sodium heparinized venous blood (collected *via* the VAP) were immediately incubated with either 0, 100 or 1000 ng/mL LPS for four hours (see **Supplementary Methods** for further details). Samples were then centrifuged at 2000 xG and the supernatant stored at -80°C and TNF-α analyzed as described in **Supplementary Methods**.

###### Flow Cytometry

Sodium heparin vacutainers were filled with 10 mL blood collected from the VAP and kept at 4°C until processing (which occurred within 2 h from collection). Blood was treated with red blood cell lysis buffer (ammonium chloride lysis buffer), then centrifuged at 2000 xG and washed twice in cold PBS. Cells were transferred to individual FACS tubes containing 900 µL FACSFlow™ buffer (342003, Becton, Dickinson UK Ltd., Wokingham, UK).

Cells were then stained for CD4/CD8, CD16/CD14 and CD172a/CD163 using dye-conjugated antibodies. Isotype matched controls were used. All antibodies are listed in **Supplementary Table S6**. The samples were analyzed on a BD FACSCalibur™ (Becton, Dickinson) and 10000 events acquired using BD CellQuest Pro™ software. Details of gating strategy are reported in the **Supplementary Materials**.

###### Hematology and Biochemistry

Samples for hematology and biochemistry analyses were submitted to a commercial laboratory. Full details of analytes measured are shown in **Supplementary Table S2**.

##### Biomarker Responses in Animals in the Endotoxemic Phase

Neuromodulation was continued following the naïve assessment described above and the next day *in vivo* LPS testing was performed. Sham animals were maintained for a matching time period. One sham animal was excluded from the *in vivo* LPS part of the study on veterinary recommendation for reasons not associated with the study.

###### In Vivo LPS and cytokines assay

Baseline blood was drawn from all animals at time 0 h and they were subsequently injected with LPS (0.025 µg/kg i.v. over 5 mins; *E. coli* as above; see **Supplementary Methods** for LPS preparation). This dose of LPS was chosen based on previous work in this group (4) and from a pilot study as outlined below. Following LPS injection, bloods were drawn throughout the day for cytokine analysis, hematology, biochemistry, flow cytometry and Specialized Pro-Resolvin Mediator analysis (see **Supplementary Methods**).

LPS injection was coordinated such that stimulation animals (n=6) received scheduled neuromodulation immediately before LPS injection, followed by an additional manual stimulation five minutes post-injection.

For cytokine analysis, venous blood samples collected in EDTA tubes were immediately centrifuged at 2000 xG for 5 mins at 4°C. Plasma was then separated and immediately frozen on dry ice and subsequently stored at –80°C. These plasma samples were used to measure TNF-α concentration using the commercially available ELISA kits described above (R&D systems).

All other blood analyses were run as described above.

###### Targeted Lipid Mediator Profiling

Specialized Pro-Resolving Mediator analysis was performed on plasma samples taken as above during the endotoxemic phase in this conscious study. Additionally, similar analysis was performed on plasma samples collected during a low dose anesthetized endotoxemia study with acute SpNS (4) at 0, 0.5 and 2 h post LPS administration.

Plasma lipid mediators were extracted using solid-phase extraction columns as described in (26) and **Supplementary Methods**.

#### Data Analysis

TNF-α, hematology bloodwork and flow cytometry data were analyzed in InVivoStat 4.0 (see https://invivostat.co.uk/) and visualized in Graphpad Prism 8.4.2.

##### TNF-α Data Analysis: Ex Vivo LPS Assay

The *ex vivo* TNF-α data were analyzed for each LPS concentration using a 2-way repeated measures mixed model approach, with neuromodulation as the treatment factor, timepoint (average of baseline, +2 and +7 days) as the repeated factor, and cohort as the blocking factor. The responses were log_10_ transformed prior to analysis to stabilize the variance. Planned comparisons were then made at each timepoint, and the unadjusted p-values were corrected using Hochberg’s multiple comparison procedure.

##### TNF-α Data Analysis: In Vivo LPS Assay

The area under the curve (AUC) was calculated for plasma TNF-α between 0.5 and 2.0 h post-LPS injection (AUC_0.5- 2.0_) for each animal individually; the time period during which the majority of the TNF-α increase, peak and subsequent decline occurred. Baseline for AUC for each animal was taken as the value of its baseline TNF-α prior to LPS injection. Data were analyzed using a 1-way ANCOVA approach, with neuromodulation or no neuromodulation as the treatment factor and baseline TNF-α (prior to LPS injection) as the covariate. The responses (AUC and baseline TNF-α) were log_10_ transformed prior to analysis to stabilize the variance. The cohort was included as a blocking factor to account for day-to-day variability.

##### Hematology and Biochemistry

The data were analyzed using a 2-way repeated measures mixed model approach, with neuromodulation as the treatment factor, timepoint (average of baseline, +2 and +7 days or 0-24 h for naïve and endotoxemia phases respectively) as the repeated factor, cohort as the blocking factor and baseline cell count as the covariate. The responses and covariate were log_10_ transformed prior to analysis to stabilize the variance. Planned comparisons were then made at each timepoint, and the unadjusted p-values were corrected using Hochberg’s multiple comparison procedure.

##### Flow Cytometry

The data were analyzed as per the hematology and biochemistry but without a covariate or transformation. Data were expressed as % change over the baseline value. Baseline value for the naïve phase was defined as the average value across the 3 days (–2, –1, 0) prior to initiation of stimulation. For the endotoxemia phase the baseline was defined as time 0 h prior to LPS challenge.

##### Lipid Mediators

Lipid mediators were analyzed by multivariate analysis performed using online open access metaboloanalyst (https://www.metaboanalyst.ca/MetaboAnalyst/home.xhtml) using statistical analysis tool. Features with a constant or single value across samples were deleted. Partial Least Square Discriminant Analysis was then performed following auto-scaling (mean-centered and divided by the standard deviation of each variable). Network analyses was performed on normalized concentrations (expressed as the fold change) of the lipid mediators from the Sham and SpNS groups and lipid mediator biosynthetic networks were constructed using Cytoscape 3.7.1.

#### Terminally-Anesthetized Procedures

Following *in vivo* LPS testing, 10 of 12 animals were terminally anesthetized for contrast angiography, SpNS and gross and histological pathology analysis. Two animals were excluded from terminal studies for use in further work (not described in this paper). Following anesthesia contrast angiography was used to show retention and alignment of the lead electrode around the splenic artery (see **Supplementary Information**).

Open laparotomy surgery (as described above) was performed in all animals and splenic neuromodulation performed with the IPG and an EPG. Sham animals did not undergo terminal stimulation in order to preserve their status as sham animals to provide samples for studies not described in this paper. Thresholds and stimulation response curves for changes in SpA BF and mABP were determined using parameters as described in **Supplementary Table S5** and methods as described above for intraoperative stimulation. The relationship between these thresholds and cytokine response in the *in vivo* TNF-α assay were determined within-animal.

Following measurement of BP and BF, a small SpN fascicle was dissected free from the NVB, more distal to the implanted lead cuff. eCAP recordings were in all animals to provide additional electrophysiological evidence of functional SpN fascicles.

Following euthanasia the following areas were grossly examined in detail: the abdominal wall and abdominal cavity immediately surrounding the implantation site (including the pancreas, spleen and liver); the IPG location and the cuff electrode location. Following gross pathology examination, tissues were harvested and processed for histopathology and assessed as described in the **Supplementary Methods** and **Supplementary Table S7**.

### Pilot Study Investigating LPS Dose in Non-Surgical Sham Animals

A pilot study was performed in 4 Berkshire pigs (sex, age and size matched to above study) to inform the LPS dose for the *in vivo* challenge in this paper. Additionally, they provided a group of non-surgical sham animals for comparison to animals implanted with electrodes and IPGs in the main study. Briefly, animals were anesthetized, a VAP implanted and then recovered (as described previously). *In vivo* LPS and cytokine assays were performed (as above) to assess the effect of two LPS dosages: 0.0025 µg/kg i.v. and 0.025 µg/kg i.v. The dose of 0.0025 µg/kg did not evoke TNF-α release (data not shown) unlike the 0.025 µg/kg dose which provided a robust, consistent cytokine response in the absence of any clinically adverse effects on the animals. On this basis, the 0.025 µg/kg dosage was selected for the main study.

## Results

### Refinement of Stimulation Parameters

Therapeutic SpNS should be delivered in such a way that it maintains action potential transmission and achieves continued neurotransmitter (i.e., NA) release at the terminals while minimizing local vascular effects on the splenic smooth muscle and systemic circulation. Therefore, stimulation parameters were initially refined by measuring evoked compound action potentials (eCAPs) and physiological parameters during SpNS in terminally-anesthetized pigs.

**Figure 1:**
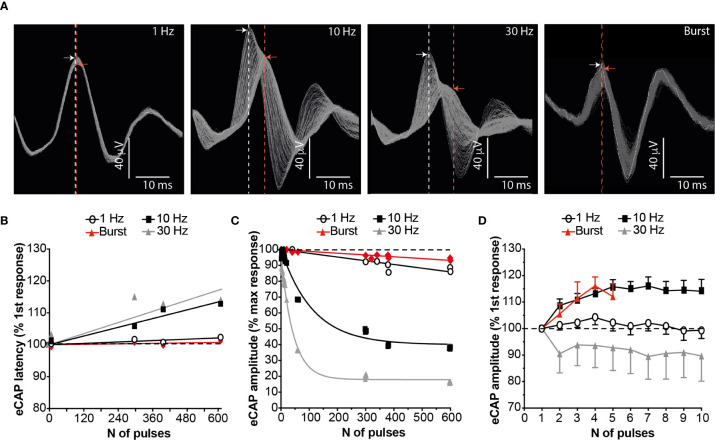
Burst Stimulation (10 Hz; 0.5s on 4.5s off) Sustains SpN Activation. **(A)** Representative SpN eCAPs obtained when stimulating the SpN at 1, 10, 30 Hz or 10 Hz burst (5 pulses delivered at 10 Hz, with a 4.5 s delay between bursts). Each image shows 300 consecutive pulses overlapped. The stimulation artefact was removed for clarity. The dashed lines indicate the latency of the peak of the first (white line) and last (red line) recorded eCAP. The arrows indicate the amplitude of the peak of the first (white arrow) and last (red arrow) recorded eCAP. **(B)** Quantified eCAP amplitude (expressed as percentage of the max response) over 600 consecutive pulses with the different pattern of stimulations shown. Data are shown as mean (n = 3). Least-squares regression curves were fitted against the data. **(C)** Quantified eCAP latency (expressed as percentage of the first response) over 600 consecutive pulses with the different pattern of stimulations shown in **(A)**. Data are shown as mean (n = 3). Least-squares regression curves were fitted against the latency data. **(D)** eCAP amplitude (expressed as percentage of the first response) over the first 5-10 pulses with the different pattern of stimulations.

There was a frequency-dependent effect of stimulation on SpN conduction velocity and eCAP amplitude. When stimulating at 1 Hz, conduction velocity was calculated as 0.84 m/s (range: 0.39 to 1.59 m/s) in line with previous data (4) and e.g (27). Continuous stimulation at frequencies greater than 1 Hz resulted in marked action potential conduction velocity slowing, as measured by increased eCAP latency; this phenomenon was evident when stimulating at 10 or 30 Hz (**Figures 1A, B** **)**. In parallel, continuous stimulation at these frequencies showed a concomitant reduction of the eCAP amplitude (**Figures 1A, C** **)**. These phenomena were almost completely prevented by stimulating at 1 Hz or by applying bursts of stimulation at 10 Hz (repeated bursts of 5 pulses at 10 Hz, 0.5 s on and 4.5 s off). During stimulation with either of these paradigms over 600 stimulation pulses, reduction of the eCAP amplitude and conduction velocity was avoided (**Figures 1A–C**). Furthermore, when stimulating with the 10 Hz burst paradigm, the conduction velocity and the eCAP amplitude actually increased between the 1^st^ and the 10^th^ pulse of a 10 Hz stimulus train, peaking after 4-6 pulses (**Figure 1D**). These initial augmentative effects were absent when using 1 or 30 Hz stimulation.

**Figure 2:**
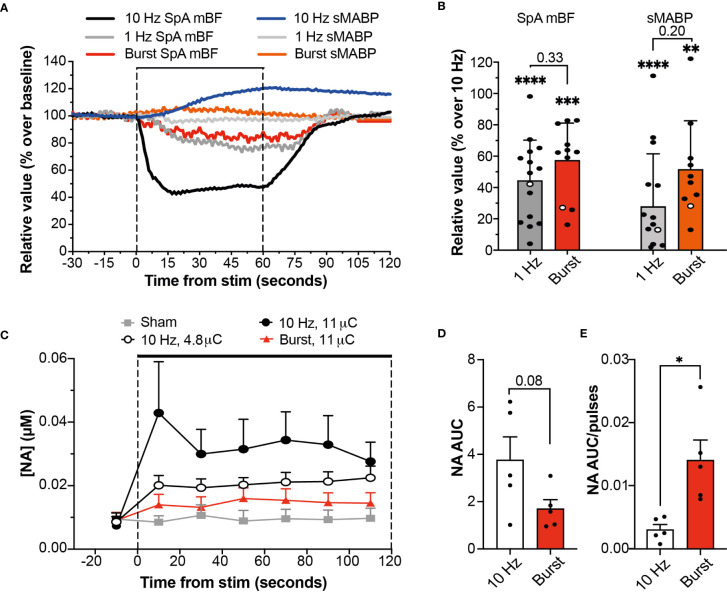
Burst Stimulation (10 Hz; 0.5s on 4.5s off) Sustains NA Release While Reducing Cardiovascular Effects. **(A)** Changes in SpA BF and mABP during a 60 s stimulation of the SpN using a 5 µC stimulus delivered at 10 Hz, 1 Hz or 10 Hz burst stimulation. Data are from stimulations within the same representative animal (shown as empty circles in 2B). **(B)** Relative SpA BF and mABP changes recorded during a 60 s stimulation delivered with stimulation at 1 or 10 Hz continuous or 10 Hz burst. Values are expressed as percentage of the average maximum change obtained at 10 Hz continuous across all animals. Individual data points are shown (at least 2 replicates from each animal) and mean (n = 4) + s.d. * represent significant statistical difference from 10 Hz continuous. **(C)** Graph showing the concentration of NA at each time point during baseline and stimulation periods (2 min) at different amplitude (0-11 µC) or pattern (continuous or burst at 10Hz) as indicated. Data are shown as mean (n = 5) + s.e.m. **(D, E)** Graph showing the comparison of NA release during stimulation at 10 Hz continuous or 10 Hz burst at the same stimulation intensity, expressed as AUC **(D)** or as AUC normalized over the total number of pulses **(E)** delivered during the 120 s period of stimulation (1200 vs 120, respectively). Statistical analysis was performed using One-way ANOVA and Tukey post-hoc correction for multiple comparison. *P ≤ 0.05; **P ≤ 0.01; ***P ≤ 0.001; ****P ≤ 0.0001. or individual P-values are shown.

Our group has previously shown that SpNS delivered at 10 Hz induces changes in SpA BF and systemic mABP that are directly related to activation of efferent (toward the spleen) SpN axons (4). The effects of stimulation using either a 1 Hz continuous or 10 Hz burst paradigm were significantly lower (approximately 50% reduction) on both local (SpA BF) and systemic (mABP) physiological parameters, compared to 10 Hz continuous stimulation (P ≤ 0.01; **Figures 2A, B** **)**. There was no difference in effect between stimulation using 1 Hz continuous or 10 Hz burst.

Taken together these data suggest that 10 Hz burst stimulation may be a more refined stimulation pattern for sustained SpN neuromodulation in the presence of reduced cardiovascular effects.

The anti-inflammatory effect of autonomic neuromodulation has been shown to be dependent on the SpN, and on NA released from nerve terminals onto splenic leukocytes (4, 8, 11). Therefore, to evaluate the potential efficacy profile of burst stimulation, the concentration of NA was used as a pathway- and mechanism-relevant biomarker. Continuous or burst stimulation at 10 Hz were applied at different stimulation intensities while the output of NA from the spleen into the SpV was measured, alongside SpA BF and mABP. Stimulation with either paradigm evoked an intensity- and frequency-dependent release of NA (**Figure 2C**) which was maintained during 120 s of stimulation. Stimulation of the SpN with the burst paradigm resulted in reduced cardiovascular effects with an apparent reduction in total NA secretion when compared to 10 Hz continuous delivered at the same stimulation charge (1.70 ± 0.39 versus 3.76 ± 0.98 µM.s, a reduction of 45.2%; P=0.08, **Figure 2D**). However, when normalized over the total number of pulses delivered, burst stimulation released a significantly higher amount of NA per stimulation pulse (P=0.010; **Figure 2E**).

Taken together, these findings suggest that burst stimulation (10 Hz; 0.5 s on; 4.5 s off) is an efficient stimulation paradigm; able to evoke maintained SpN activation and NA release, in the presence of reduced effects on the SpA BF and mABP. Given that 120 s of 10 Hz continuous stimulation has been shown to be immunomodulatory in acute studies (4), it was hypothesized that burst stimulation for 4.5 mins (1/45.2% or 2.21 times longer) would result in significant immunomodulation. This paradigm and hypothesis were then assessed in chronically-implanted animals.

### Chronic Splenic Nerve Neuromodulation

#### Chronic Implantation and Intraoperative Stimulation

**Figure 3:**
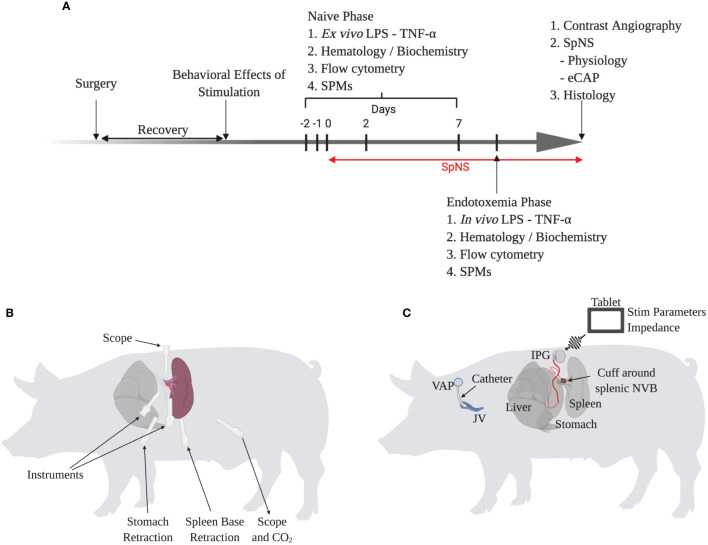
Chronic Conscious Neuromodulation Study Methods and Refinement of the Minimally-Invasive Surgical Implantation Procedure. **(A)** Schematic of overall chronic conscious study design. Each phase is described in more detail in the methods and supplementary information. **(B)** Schematic of surgical implantation procedure showing trocar position and use, relative to the spleen. **(C)** Schematic of chronic neuromodulation and blood sampling procedure. Animals were implanted with a catheter in the jugular vein and a circumferential cuff electrode around the splenic neurovascular bundle (NVB) connected to an implantable pulse generator (IPG). The IPG was wirelessly controlled by a tablet to control stimulation parameters and measure impedance.

The overall chronic study outline is shown in **Figure 3A**. A total of twelve female pigs underwent a novel minimally-invasive laparoscopic surgical procedure (**Figures 3B, C** **)**. This implantation procedure was refined through studies in both human and pig cadavers with input from human and veterinary surgeons. The splenic NVB was surgically isolated from surrounding tissue, and a circumferential cuff electrode was implanted around the artery and nerve plexus. The electrode lead then exited the abdomen where it was connected to a subcutaneously-located implantable pulse generator (IPG). A catheter was placed in the jugular vein and connected to a subcutaneously-implanted vascular access port to enable serial blood sampling.

During laparoscopic implantation of the stimulation lead and IPG, SpN neuromodulation with a 10 Hz continuous paradigm (60 s) was used to confirm both placement and function of the implanted lead, IPG and target engagement, as performed in the on-going clinical trial described previously. Animals were stimulated at their respective IPG charge outputs (see methods for details) of 15 µC or 40 µC. Stimulation evoked an increase in mABP of 9.81 ± 6.83% (n=5) at 15 µC or 10.31 ± 5.87% (n=7) at 40 µC (**Supplementary Figure S1**). In all animals the increase in mABP was temporally-correlated to the onset and offset of stimulation, in line with the acute experiments described above, and was used as confirmation of target engagement and functionality of the implanted SpN neuromodulation system prior to animal recovery.

Following implantation surgery, all animals recovered well without complications. Electrodes were monitored throughout the course of the study to assess continued function of the IPG and stimulation lead integrity.

Following implantation, impedance was 404.33 ± 62.68 Ω (n=10 of 12), and peaked around 11 days post-implantation (583.21 ± 89.78 Ω) before plateauing around 540 Ω for the remainder of the study (**Supplementary Figure S2**).

Given the practical limitations of chronically-implanted large-animal studies and associated biomarker assays, animals proceeded through the study in four cohorts, with each cohort consisting of animals receiving chronic SpN neuromodulation, and sham animals implanted with an identical stimulation lead and IPG receiving no conscious neuromodulation. Evidence of mechanical failure of the electrode was noted in two animals prior to onset of chronic neuromodulation. Given that electrode failure was not caused by animal- or nerve-related issues, these animals were assigned to the sham group. Other animals were randomly assigned to either the SpNS or sham group.

#### Effects of Chronic Neuromodulation in Naïve Animals

To assess behavioral tolerability of SpN neuromodulation, approximately 14 days following device implantation, SpNS pigs were stimulated using the 10 Hz burst paradigm using a step-up protocol between 1 and 15 or 40 µC (in increments of 1-2 µC). The behavioral responses to stimulation were not assessed in sham animals. There was no evidence of behaviors or responses indicative of perception of neuromodulation of the SpN in any animal at any of the stimulation amplitudes used. Animals were then maintained without stimulation until approximately 28 days following device implantation.

The effects of chronic SpN neuromodulation were then assessed to examine any potential immunosuppressive effects in animals naïve to inflammatory challenge. This was performed by measuring *ex vivo* peripheral whole blood LPS-induced TNF-α production, hematological and clinical biochemistry measurements and flow cytometry. Baseline peripheral blood samples were collected from all the animals prior to initiation of chronic neuromodulation, nominally days -2, -1 and Day 0.

Chronic neuromodulation was initiated and maintained for 7 days at either 15 or 40 µC using the 10 Hz burst paradigm (5 mins/session; 12x/day; full parameters described in the methods) and further blood samples were collected on Days 2 and 7. There was a clear concentration-response relationship between LPS and the amount of TNF-α measured in the plasma isolated from whole blood incubated *ex vivo* with varying concentrations of endotoxin (**Supplementary Figures S3A–C**). Considering the neuromodulation period between day 0 and 7, there was no difference in TNF-α release between SpNS and sham animals. Additionally, there was no effect of SpNS on total white blood cell, neutrophil or monocyte counts (**Figures S3D–F** respectively), or any other hematological or clinical biochemistry parameters (**Table S1**), comparing either within the SpNS animal groups between baseline and Day 2 or Day 7, or between SpNS and sham animals on Day 2 or Day 7.

To further evaluate effects on the immune system in animals naïve to inflammatory challenge, peripheral blood leukocytes were stained with antibody against the surface molecules CD14/CD16, or CD172a/CD163, or CD4/CD8, to identify potential changes in major leukocyte subsets by flow cytometry. As expected CD14, CD16 and CD172a markers were expressed not only by monocytes but also by lymphocytes and granulocytes. Plotting these markers against side scatter allowed us to distinguish between these three populations and therefore to specifically quantify monocytes (see **Supplementary Figures S4, 5** **;** and Methods for details). Subsequent analysis did not reveal any significant effect of SpNS within or between groups when quantifying the proportion of monocytes that were CD16^+^ (**Supplementary Figures S3G, H**), CD172a^+^ (**Figure S3I, J**), CD14^+^ (**Figure S6D**) or CD163^+^ (**Supplementary Figure S6E**). No significant differences were observed when quantifying the expression (as median fluorescence intensity; MFI) of CD14 on CD16^+^ monocytes (**Supplementary Figures S3K, L**). Furthermore, no changes were noted in the T cell compartment (**Supplementary Figures S6A–C**).

#### Effects of Chronic Neuromodulation During *In Vivo* Endotoxemia

Following evaluation of the effects of SpNS in naïve animals, the effects of neuromodulation were assessed in an *in vivo* endotoxemia model. Neuromodulation was continued for a further day (day 8) following which animals were subjected to low dose endotoxemia by systemic injection of LPS *via* the jugular vein catheter. Peripheral blood was collected to quantify LPS-induced plasma TNF-α and SPM concentrations in peripheral plasma, and hematological and clinical biochemistry parameters.

**Figure 4:**
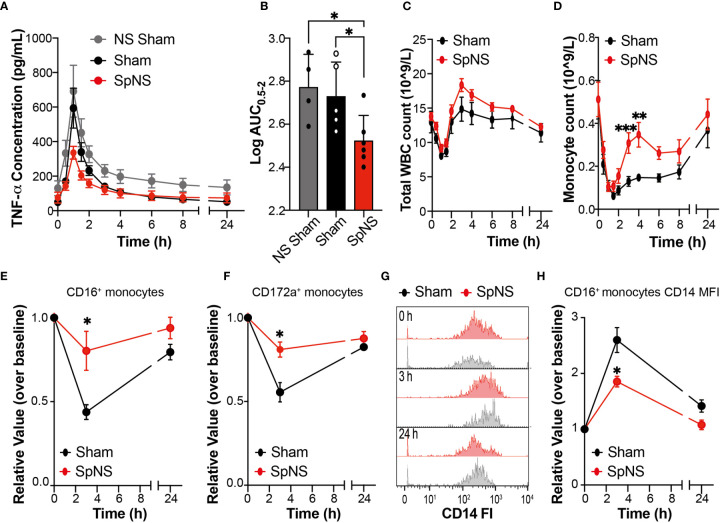
Chronic SpN Neuromodulation Evokes Anti-Inflammatory Effects During LPS-Induced Endotoxemia. **(A)** Graphs showing TNF-α concentration quantified in the peripheral plasma collected at different time points after i.v. injection of 0.025 µg/kg of LPS. The data are shown for non-surgical (NS) sham (grey), sham (black) and SpNS (red) animals. **(B)** Graph showing the quantification of the relevant TNF-α AUC (between 0.5-2 h post LPS) derived from **(A)**. Data are shown and were analyzed using the expressed as Log_10_ normalized AUC values. The single data points with mean are shown **(C, D)** Graphs showing total white blood cell **(C)** or monocyte **(D)** cell counts from peripheral blood between 0 and 24 h post LPS injection. **(E, F)** Quantification of peripheral blood monocytes stained with antibodies against CD16 **(E)** or CD172a **(F)** over time. **(G)** Representative histograms showing the changes over time of CD14 expression on CD16^+^ monocytes in a sham and a SpNS representative pig. **(H)** Quantification of the median fluorescence intensity (MFI) of CD14 expression on CD16^+^ monocytes over time. **(A, C–F, H)** Data are expressed as mean ± s.e.m. **(E, F, H)** Data are expressed as relative change over the baseline (value prior to LPS injection). **(A, B)** n = 6 for SpNS; n = 5 for sham. **(C–E)** n = 6 for SpNS; n = 5 for sham. **(E, F, H)** n = 3 for SpNS; n = 4 for sham. The sham group is shown in black and SpNS group in red. *P ≤ 0.05; **P ≤ 0.01; ***P ≤ 0.001 *vs* Sham or NS Sham.

The dose of LPS used in the current study was validated in a preliminary cohort of animals implanted only with the vascular access catheter (n=4; non-surgical (NS) sham), and was initially selected based on experience with anesthetized LPS experiments (4) and evidence in literature. The dose selected (0.025 µg/kg, i.v.) was shown to evoke a robust increase in plasma TNF-α levels (**Figure 4A**; grey trace) in the presence of only mild to moderate clinical behaviors (see Methods).

Following systemic injection of LPS, typical clinical behaviors (lethargy and shivering) were seen in all animals; these started around 45 min post-injection and persisted for around 45 min in the case of shivering and 4 h for the lethargy. These behaviors resolved without the need for medical intervention. In all pigs, LPS injection caused an upregulation of systemic TNF-α levels (**Figure 4A** **)** peaking at 1 h post injection. However, this dynamic upregulation of TNF-α was smaller in SpNS animals in comparison to sham as well as NS sham animals. Quantification of the total amount of TNF-α, measured as area under the curve (AUC) between 0.5 and 2.0 h post-LPS injection (the time period including TNF-α increase, peak and subsequent decline occurred), revealed a significant reduction of TNF-α in SpNS vs sham animals (**Figure 4B**); there was a reduction of approximately 40% in SpNS vs sham animals (P=0.029; 345.0 ± 42.62 vs 566.0 ± 213.0 pg/ml.h, respectively). The amount of TNF-α was also significantly reduced compared to NS sham (P=0.046), while no significant difference was found between sham and NS sham animals.

Analysis of peripheral circulating leukocytes showed LPS-induced dynamic changes in total WBC (**Figure 4C**), neutrophils (**Supplementary Figure S7A**), monocytes (**Figure 4D**) and lymphocytes (**Supplementary Figures S7F–H**). In all animals there was a significant effect of time in all the four parameters (P<0.0001 for all). Total circulating WBCs showed an initial leukopenia, followed by leukocytosis. There was approximately a 40% reduction from baseline values of WBCs which reached a minimum around 1 h, followed by an elevation at 4 h (130-160% of baseline) which resolved to baseline values by 24 h (**Figure 4C**). These changes were mainly driven by variation in neutrophil number (**Supplementary Figure S7A**). Additionally, monocytes showed an initial reduction, with a minimum around 1.5 h, which then resolved by 24 h (**Figure 4D**). When comparing SpNS and sham animals, there was a significant effect of neuromodulation on the number of monocytes at 3 and 4 h post-LPS (overall ANCOVA P=0.021; post-hoc analysis P=0.004 and P=0.009 respectively), with a higher proportion of circulating monocytes in SpNS animals versus sham. There was no significant effect of SpNS on WBCs or neutrophils. Furthermore, there were no changes in blood biochemistry parameters following LPS administration in either SpNS or sham animals. All hematological and clinical biochemistry data can be found in **Supplementary Table S2**.

Flow cytometry analysis also revealed an effect of LPS administration on the proportion of peripheral leukocyte subsets at 3 h post LPS, before returning toward baseline levels at 24 h (in line with hematological analysis). There was however a significantly smaller LPS-induced reduction in circulating monocytes in SpNS animals, quantified as CD16^+^ monocytes (P=0.039), with post-hoc analysis revealing a significant effect at the 3 h timepoint (P=0.031; **Figures 4E** and **S7B**). Analysis of CD172a^+^ monocytes revealed no overall significant effect of SpNS (P=0.065), however post-hoc analysis revealed a significantly smaller LPS-induced reduction at the 3 h timepoint in stimulated animals (P=0.029; **Figure 4F** and **S7C**). Smaller changes from baseline in stimulated animals were also seen for CD14^+^ monocytes and CD163^+^ monocytes (**Figures S7D–E**). Additionally, when assessing the expression of CD14 on CD16^+^ monocytes, stimulated animals presented with a reduced CD14 expression (MFI) level (**Figures 4G, H** **;** overall ANCOVA P=0.047; post-hoc analysis P=0.040 at the 3 h timepoint); no difference was observed for CD16^+^CD14^low^. No statistically significant differences between groups were observed for CD4^+^ and CD8^+^ lymphocytes (**Supplementary Figure S7F–G**).

#### Effects of Chronic Neuromodulation on Specialized Pro-resolving Mediators

**Figure 5:**
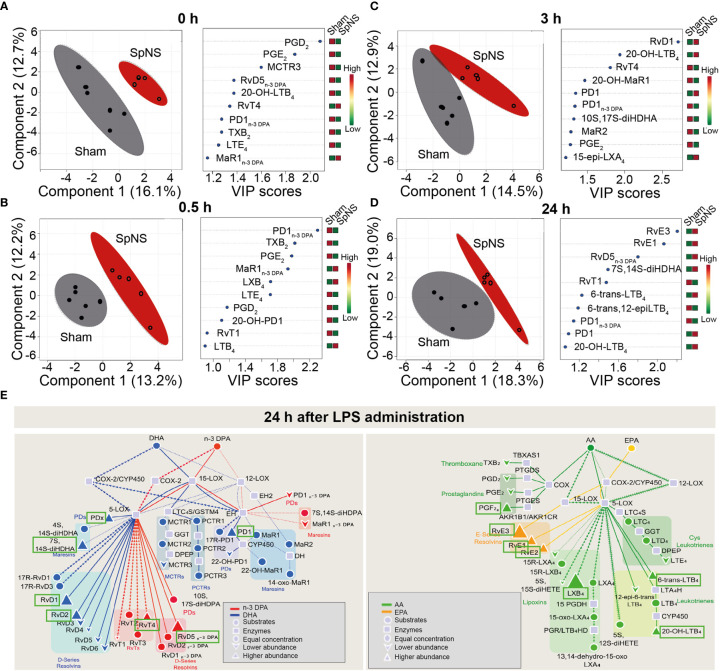
Chronic SpN Neuromodulation reprograms peripheral blood lipid mediator profiles. Porcine splenic nerve was stimulated chronically for 8 days. Plasma was collected immediately prior to LPS challenge **(A)**, 0.5 **(B)**, 3 **(C)** and 24 **(D)** h post LPS challenge and lipid mediators were investigated using LC-MS/MS-based profiling. Results were interrogated using Partial Least Square Discriminant Analysis. (Left panels) Display Score Plots. Colored area represents the 95% interval confidence. (Right panels) Plots displaying the lipid mediators with the 10 highest VIP scores from component 1. n = 5 for Sham group and n = 6 for SpNS group. **(E)** Flux down each of the bioactive metabolomes was assessed in the samples collected 24h post LPS injection. Pathway analysis for the differential expression of mediators from the DHA and n-3 DPA (left panel) and EPA and AA (right panel) bioactive metabolomes in SpNS group when compared to Sham group. Results are expressed as the fold change. Green boxes indicate the SPMs upregulated in the SpNS group at 24h post LPS (vs Sham). n = 5 for Sham group and n = 6 for SpNS group.

In order to understand if SpN neuromodulation could affect inflammation resolution processes, further than modulating cytokine production, the concentration of SPMs in peripheral plasma was quantified using LC-MS/MS-based lipid mediator profiling. Lipid mediators from all four major essential fatty acid metabolomes were identified, including the docosahexaenoic acid and n-3 docosapentaenoic acid metabolomes (**Supplementary Table S3**). These mediators were detected in accordance with published criteria that include matching retention time and at least six diagnostic ions in the MS/MS spectrum (26). Assessment of plasma mediator concentrations using partial least square discriminant analysis (PLS-DA) demonstrated that chronic SpN neuromodulation led to a shift in plasma lipid mediator profile. This was highlighted by a shift in the cluster of lipid mediators representing plasma collected from pigs subjected to chronic neuromodulation when compared with sham animals (**Figures 5A–D**). Of note, this shift in plasma lipid mediator profiles was present at 8 days after initiation of chronic neuromodulation (**Figure 5A**), prior to LPS administration, and was retained throughout the 24 h time course of LPS challenge (**Figures 5B–D**). In order to evaluate the lipid mediators that contributed to this separation between the two experimental groups, the variable in importance (VIP) score was assessed; this determines the contribution of each mediator to the observed group separation. Several pro-inflammatory eicosanoids including PGD_2_ and PGE_2,_ as well as SPMs from the protectin and resolvin bioactive metabolomes, were among those that contributed to the separation between SpNS and sham animals (**Figures 5A–D**; right panels). Of note, concentrations of the inflammatory eicosanoids were decreased in the stimulated group, whereas SPM concentrations were increased (**Figures 5A–D**; and **Supplementary Table S3**).

Lipid mediator pathway analysis was conducted by assessing the flux down each of the four bioactive metabolomes, comparing between SpNS and sham pigs at 24 h post LPS. An upregulation of several ALOX15-derived mediators, including the DHA-derived RvD1, RvD2, PD1, the n-3 DPA derived RvD5_n-3 DPA_, the EPA-derived RvE1, RvE2, RvE3 and the AA-derived LXB_4_, was observed in SpNS pigs compared to sham (**Figure 5E**). Interestingly, upregulation of the ALOX-15 pathway was also observed prior to LPS administration (8 days after initiation of chronic neuromodulation) characterized by increased levels of family D and E resolvins (RvD1, RvD5 and RvE1, RvE3) and LXB4 (**Supplementary Figure S8**).

Finally, to determine whether SpNS stimulation *directly* regulates plasma lipid mediator profiles, rather than as a downstream consequence of chronic stimulation, the impact of *acute* SpNS on plasma SPM concentrations was investigated using samples collected from experiments previously performed under terminal anesthesia (4). In line with chronic stimulation experiments, the lipid mediator profiles from acutely SpN stimulated pigs (stimulation applied continuously from -2 to +1 h relative to LPS administration) clustered separately from those obtained from sham animals when using PLS-DA across 2 h time window after LPS administration (**Supplementary Figure S9**). Assessment of VIP scores demonstrated that acute SpNS downregulated plasma prostaglandin concentrations, and upregulated resolvin and protectin concentration (**Supplementary Figures S9A–C**; right panels; and **Supplementary Table S4**). Similarly to chronically stimulated pigs, acute stimulation promoted SPM profile separation prior to LPS injection (2 hours after initiation of stimulation) mainly *via* upregulation of a number of ALOX15-derived SPMs (including RvD3 and RvE3) at both 0 h (**Supplementary Figure S10**) and 2 h (**Supplementary Figure S11**) after LPS injection.

#### Terminal Assessment of Electrode-to-Nerve Integrity

Following *in vivo* LPS assessment, animals were terminally anesthetized between 40- and 70-days post-implantation and underwent procedures to confirm retention of the stimulation electrode around the splenic NVB and to confirm continued SpN function through stimulation-mediated target engagement (increase in SpA BF and mABP) and subsequent post-mortem histological examination.

Contrast angiography of the SpA demonstrated that all the electrodes were retained around the splenic NVB (**Supplementary Figure S12A**). Successively the animals were instrumented to perform electrophysiological recordings. To evaluate the functionality of the nerve-to-spleen circuit, multiple SpN stimulations were performed while recording evoked changes in systemic mABP, SpA BF and eCAP.

**Figure 6:**
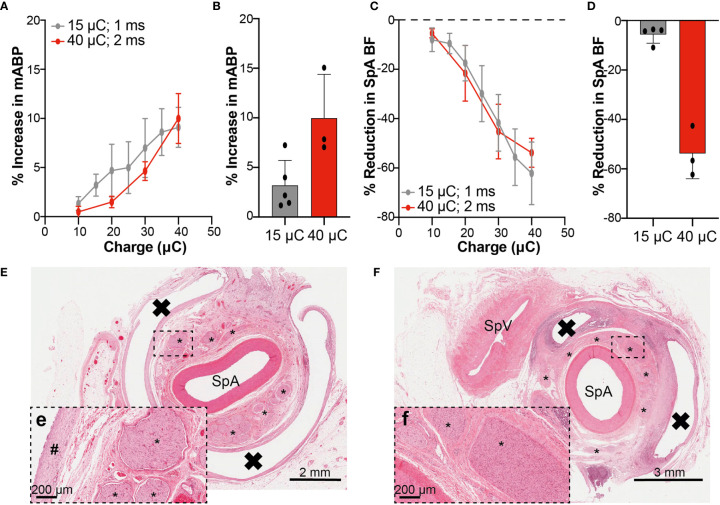
Physiological and Histological Assessment Confirms Integrity of Electrode-Nerve-Spleen Circuitry at Termination. **(A, C)** Percentage changes (normalized to pre-stimulation baseline) in mABP **(A)** and SpA BF **(C)** obtained when stimulating the SpN with the implanted cuff at different intensities, using an external stimulator, up to 40 μC. The curves obtained with two different pulse widths (1 and 2 ms) are shown. **(B, D)** Graphs showing the maximum percentage changes in mABP and SpA BF achieved at termination at relevant IPG output (15 or 40 μC), delivered at 10 Hz continuously for 60 s. Data in A-D are shown as mean ± s.e.m. **(E)** Low power image of transverse section from the splenic artery from a representative animal. The SpA is surrounded by numerous fascicles with normal morphology. The white band encircling the artery and fascicles represents the former location of the implanted cuff. The black box highlights the region represented in (E-e; higher power image) and shows nerve fascicles with normal morphology. A band of mature fibrotic tissue is part of the fibrotic capsule surrounding the implant and represents an expected and acceptable response to the device. **(F)** Low power image of a transverse section from the splenic artery from a second representative animal. A more significant tissue response to the presence of the implanted cuff is observed here when compared to that seen in **(E)**. The SpA is surrounded by numerous fascicles. The white band surrounding segments of the artery and fascicles represents the former location of the implanted cuff. The black box highlights the region represented in (F-f; higher power image) and shows nerve fascicles with evidence of increased cellularity (Schwann cell hyperplasia) and perineural fibrosis and represents a localized chronic response to low level nerve injury. * = Nerve fascicle; # = mature band of fibrotic tissue, X = former location of implanted cuff, SpV, splenic vein.

eCAP generation, confirming the integrity of the cuff electrode-to-nerve interface, was demonstrated in all animals in recordings made distally to the cuff electrode (**Supplementary Figure S12B**). Stimulation of the SpN (at the amplitude delivered during conscious phase) with a 10 Hz continuous paradigm for 60 s evoked an increase in mABP of 3.20 ± 2.51% and a decrease in SpA BF of -5.68 ± 3.50% at 15 µC (n=5; **Figures 6B, D**). At 40 µC (**Figures 6B, D**) changes in mABP and SpA BF were 9.98 ± 4.41 and -53.84 ± 10.15% respectively (n=3). Full stimulation-response curves were constructed with stimulation *via* an external pulse generator up to 40 µC using a 1 or 2 ms pulse width (**Figures 6A, C**). At 40 µC, a similar magnitude in response was observed in both groups of animals with an increase in mABP (1 ms: 9.10 ± 4.07%; 2 ms: 9.98 ± 4.41%) or decrease in SpA BF (1 ms: -62.15 ± 25.41; 2 ms: 53.84 ± 10.15%).

Considering change in mABP or SpA BF at termination as an indication of nerve engagement during the conscious phase of the study, **Supplementary Figures S12C–D** show the correlation between these measurements and the TNF-α AUC_0.5-2.0_. Comparing across all animals, there was no apparent correlation between the degree of nerve engagement (as assessed by change in mABP or SpA BF) and the overall TNF-α AUC (r^2^ = 0.04 or 0.17 respectively).

Finally, all animals were heparinized and then euthanized. Gross pathological and histopathological examinations were then performed. No gross pathological abnormalities were noted in any animal. Histopathological analysis demonstrated that leads were well-tolerated, with no changes observed in SpA or vein. In some animals, changes in nerve fascicles restricted to Schwann cell hyperplasia and perineurial fibrosis with occasional fascicles showing evidence of intra-fascicular edema were observed. Representative histological sections from two SpNS animals show minimal or low severity (**Figures 6E, F**, respectively) response to chronic cuff implantation around the splenic NVB and stimulation, further confirming the integrity of the functional neural connections. Furthermore, no device-related abnormalities were observed in tissue sections from the spleen, pancreas or liver.

## Discussion

The results presented here using a large-animal model provide: i) a novel preclinical surgical procedure to implant a SpN circumferential cuff electrode and IPG; ii) a refinement of SpN stimulation parameters for efficacy and target engagement; and iii) a robust demonstration of the immunomodulatory effects of SpN neuromodulation (induction of pro-resolutive SPMs, suppression of LPS-induced TNF-α levels, and monocyte mobilization and activation).

Immunomodulatory effects were achieved utilizing refined stimulation parameters that were well tolerated by the animals (up to 40 µC) and did not affect nerve integrity (assessed between 40 and 70 days post-implantation), as demonstrated by terminal electrophysiology, physiology and histology. These are fundamental requirements of bioelectronic medicines. Ideal stimulation parameters, in fact, should be able to i) maintain nerve engagement over time, ii) produce efficient neurotransmitter release, iii) cause minimal/no off-target effects, iv) cause no/minimal axonal and tissue damage, while iv) maintaining treatment efficacy. Ideally, this should be demonstrated in an animal model sized to best match the anatomy and physiology of the human patient (20, 28). Recently we have shown that for splenic neuro-immune modulation the pig represents such a human-relevant and functional model (4).

Across species, the SpN consists mainly of unmyelinated axons (4, 29), which represent a particular challenge in the field of neuromodulation. Achieving and maintaining optimal unmyelinated nerve engagement is a difficult requirement due to the higher activation thresholds (30) when compared to myelinated nerves. Uniform activation of the SpN is further challenging due to the structure of the splenic NVB composed of an artery, associated adipose tissue, and dispersed fascicles (31, 32). When stimulating at 1 Hz, conduction velocity was in line with previous data for unmyelinated axons (4) and e.g (27). Stimulation of the SpN at continuous frequencies higher than 1 Hz (specifically 10 and 30 Hz) caused an increase in eCAP latency as well as a reduction in eCAP amplitude, suggesting a reduction in axonal recruitment and as a result, less efficient nerve activation over successive stimulation pulses. This is again in line with that previously described for other unmyelinated axons. Stimulation of sensory nerves, for example, is known to cause “action potential conduction slowing” (27, 33) where repetitive stimulations cause progressive reduction of conduction velocity (as evidenced by an increase in latency) and increase in activation threshold (27). Therefore, low frequency stimulation may be used to efficiently stimulate the SpN. However, stimulation at higher frequencies also allows the delivery of larger amounts of neurotransmitter (i.e. NA) within a certain time frame, potentially producing a more robust immunomodulatory effect. Interestingly, when the SpN was stimulated continuously at 10 Hz, during the initial period (~10 pulses) an augmentative effect was observed on eCAP recordings (increase in eCAP amplitude and conduction velocity) followed by subsequent slowing and reduction of the eCAP. The former effect, named “action potential conduction speeding” has also previously been observed in unmyelinated sensory nerves (34). Together these suggest that highly efficient stimulation can be achieved at frequencies higher than 1 Hz, providing that a short “on” period is used. This is in line with our data showing that 10 Hz burst (i.e. 0.5 s on; 4.5 s off) provided efficient nerve recruitment (increase in eCAP amplitude and no conduction slowing) and a more efficient release of NA (when normalized to the total number of pulses delivered in the same time window). This theoretically translates into a more energy-efficient clinical therapy; with reduced total stimulation on-time of the neuromodulation device. Additionally, and perhaps more importantly, stimulation with the 10 Hz burst paradigm also reduced physiological effects on mABP and SpA BF, when compared to continuous 10 Hz (or higher) stimulation, to levels comparable to those caused by low frequency (1 Hz) stimulation.

While continuous stimulation at 10 Hz (or higher) is not preferred for long-term chronic use, due to the local and systemic cardiovascular effects, it still provides a useful paradigm for intraoperative assessment of nerve target engagement (4). SpNS-induced increase in systemic blood pressure seen in the pig is due to smooth muscle-mediated splenic contraction, with consequent release of stored blood and vasoactive factors (4, 35). The magnitude of this phenomenon in man is currently unclear since reports on splenic contractions are sparse and discordant (36, 37). Changes in SpA BF induced by activation of SpN represent a potential acute biomarker that is currently under investigation in human clinical trials (clinicaltrials.gov Identifier: NCT04171011). However, minimizing the impact of any chronic neuromodulation therapy on the cardiovascular system is desirable for long-term human use. The use of the burst stimulation paradigm will allow the clinical investigation of the efficacy of long-term SpN neuromodulation, in the presence of reduced effects on the cardiovascular system, whilst maintaining nerve activation and neurotransmitter release in the spleen.

Importantly for translation to human use, stimulation of the SpN resulted in no observable sensation in conscious animals. Using the burst stimulation parameters there was no evidence of behavioral changes during SpN neuromodulation up to 40 µC. This is in line with other work showing that only a small proportion (<5%) of SpN axons in pigs and humans are afferent in phenotype, based on calcitonin gene-related peptide staining (4, 31). Furthermore, literature evidence of splenic afferent fibers has described roles only associated with mechanical and pressure sensing (38). Together these findings suggest that SpN neuromodulation may be equally well tolerated in man, but it remains to be confirmed in future clinical trials.

Based on the NA output measured during acute terminally-anesthetized experiments we hypothesized that a five-minute stimulation with the 10 Hz burst paradigm would induce immunomodulatory effects. Stimulation of the SpN in conscious pigs showed a clear suppression of peripheral TNF-α in the systemic circulation following systemic LPS-challenge, in line with the results obtained in similar models used in anesthetized mice, rats and pigs (2, 4, 8). In this study, however, the stimulation resulted in a stronger (ca. 50%) reduction as compared to that previously observed by our group in pigs under terminal anesthesia (ca. 20-30%) (4). This could reflect either an unmasked effect of stimulation in a setting devoid of anesthetic agents, known to affect the nervous and immune system, or a cumulative immunomodulatory effect due to the delivery of multiple stimulations over several days when compared to acute stimulation.

The immunomodulatory effect of splenic nerve stimulation is most likely caused by activation of efferent nerve fibers, rather than a reduction in SpA BF. The previous study by Donegà et al. (4) compared SpNS versus left vagus nerve stimulation (LVNS; included as a positive control) versus sham (mechanical partial occlusion of the splenic artery resulting in a 50% reduction in blood flow) in a terminally-anaesthetized LPS-challenge model in pigs. There was a significant inhibitory effect of SpNS or LVNS on hypotension, tachycardia and ultimately survival. However there was no effect of mechanical occlusion of the splenic artery in sham animals. Additionally it was demonstrated that NA is able to suppress TNF-α release *in vitro* in pig splenocyte cultures.

The terminal physiological measures allowed us to estimate the degree of SpN activation evoked in the animals during the chronic therapeutic stimulation. Using the previously described correlation between SpA BF changes and SpN activation (4), we estimated that the therapeutic stimulation intensity delivered throughout conscious chronic neuromodulation evoked up to 60% nerve activation across all animals tested. Interestingly, there was no apparent evidence of correlation between the magnitude of SpA BF response measured at termination (as a surrogate indication of the degree of nerve engagement throughout the study) and the magnitude of the immunomodulatory effect (based on TNF-α AUC_0.5-2.0_). A similar activation level led to cytokine suppression in our previous terminal studies (4), suggesting that immunomodulatory activity in response to exogenously-applied LPS can be achieved without requiring 100% nerve activation. Clinical studies will help determine the clinical translation of this phenomenon in patients with acute and/or chronic inflammatory disorders. Although this observation may vary among different inflammatory conditions, it represents an important precedent in the case that tolerability to nerve activation (however unlikely) would limit the amplitude of each individual stimulation in certain patients.

The data presented here also extend our understanding of the immunomodulatory effect induced by SpN neuromodulation, beyond regulation of cytokine production. Modulation of TNF-α production by SpN stimulation was associated with a different response in peripheral monocytes when compared to sham animals. Endotoxin administration is known to cause recruitment of peripheral leukocytes into marginal pools, with consequent reduction of circulating levels (39), as observed here. However, SpN stimulated animals showed a less pronounced change in peripheral monocytes following LPS injection, as measured by hematology and flow cytometry. In particular, CD163^+^ and CD172a^+^ monocytes were more strongly reduced in sham animals at 3 h post-LPS. These populations in the pig are the closest representation of the human (CD14^dim^CD16^+^) and mouse (CX3CR1^high^Gr1^-^CCR2) “non-classical monocytes” (40–42). These monocytes have been shown in mice to patrol blood vessels and rapidly infiltrate tissues during inflammation where they produce cytokines and chemokines (43), and have been associated with resolution functions (44). In parallel we also observed a reduced accumulation of CD16^+^CD14^high^ monocytes in stimulated animals, which in pigs represent the population of “classical monocytes”, characterized in humans by CD14^high^CD16^-^ and in mice by CX3CR1^low^Gr1^+^CCR2^+^ (40, 43, 45, 46). These monocytes are known instead to be slowly activated in response to inflammatory stimuli and then infiltrate inflamed tissues and produce pro-inflammatory mediators (43). Taken together these data suggest that SpNS reduces the monocyte response to LPS, resulting in a reduction of classical pro-inflammatory monocyte peripheral accumulation (CD16^+^CD14^high^) and an increased (compare to sham) proportion of non-classical, pro-resolving monocytes (CD163^+^, CD172a^+^). This effect could be directly mediated by SpNS, possibly affecting monocyte-endothelium interactions and cell recruitment, or as a cascade consequence of the modulation of cytokine (and other mediators) production.

In addition to reducing classical pro-inflammatory responses, SpN neuromodulation led to the peripheral accumulation of specialized pro-resolutive lipid mediators able to influence leukocyte responses. Recent literature suggests that vagal connections to abdominal organs may be key in regulating resolution mechanisms (23, 24). For the first time we show that the SpN may be part of this circuitry and that acute and chronic stimulation can modulate SPM production. The observed upregulation of ALOX15-derived SPMs in stimulated animals suggests that the splenic nerve may be regulating specific mechanisms in resolution processes.

The endotoxemia model used here is known to activate immunological pathways characteristics of many inflammatory diseases and therefore we infer SpN neuromodulation may have beneficial effects in inflammatory conditions, in particular where monocytes and splenic leukocytes play a crucial role in the pathogenesis and maintenance of disease activity. In addition, pro-resolving SPMs are known to play a crucial role in pre-clinical models and patients with acute and chronic inflammatory conditions, such as RA, atherosclerosis and myocardial infarction. For example, some of the ALOX-15 derived SPMs specifically regulated by SpNS, like RvD3 and RvD1, have been shown to have therapeutic effect in arthritis models and to be produced by macrophages abundant in drug-free remission patients (47, 48); RvD2 and MaR1 prevent atheroprogression in mice (49); RvD1 produced by spleen-derived leukocytes drives resolution in the heart after infarction (50). In line with the above, early evidence exists that modulating SpN activity in mice with arthritis can reduce disease activity (10).

While the SpN stimulation directly targets the spleen, it is of note that changes were observed in the systemic circulation. Specifically, both acute or chronic stimulation were able to modulate the expression of SPMs, with most changes associated with ALOX15-derived metabolites, in either naïve or endotoxemic states. These findings suggest that the ALOX15 pathway is specifically activated by SpN neuromodulation. It is currently unknown if this effect in driven directly within the spleen. For example, outflow of NA and other factors from the spleen may potentially cause downstream effects on peripheral cells and other organs. However, it is known that NA secreted from the spleen into peripheral circulation is rapidly removed by circulating metabolizing enzymes and should have very little effect on other organs/cells. Indeed, in a previous study following SpN neuromodulation, we observed an increase in plasma NA only in blood sampled locally from the splenic vein and not from the systemic circulation (4). In addition, during 7-days of stimulation no changes in non-classical CD163^+^ or CD172a^+^ monocytes were observed. Non-classical monocytes have been shown to be mobilized from peripheral pools by systemic catecholamine release (51). Therefore, although some NA exits the spleen parenchyma, the concentration is unlikely to be sufficient to cause systemic effects. Clinical trials will examine the magnitude of effect of SpN neuromodulation on these pathway-specific mediators and their utility as a measure of target engagement. While SpA BF can be used intraoperatively, defining a chronic conscious peripheral biomarker of SpN activation of immunological pathways within the spleen would allow the clinician to optimally adjust stimulation parameters to maximize therapeutic effect while minimizing off-target effects.

Importantly, prior to LPS challenge we did not observe systemic immune suppression, as assessed by *ex vivo* TNF-α assays, peripheral blood hematology and flow cytometry. This strengthens our original observations in acutely-stimulated animals where no changes in peripheral white blood cells were observed following SpNS (or VN stimulation), as opposed to the leukopenia induced by pharmacological treatment with dexamethasone (4). Thus, we propose that SpN stimulation, rather than globally suppressing the immune system, is able to prime it toward a more balanced and pro-resolutive phenotype that translates into reduced inflammatory responses upon endotoxin challenge.

This study also investigated the outcomes of chronic implantation of a circumferential cuff electrode around the splenic NVB and any effects of chronic neuromodulation by such a device. The laparoscopic surgical implantation procedure was developed through collaborative work between veterinary and human surgeons and stimulation electrode implantation was designed to be clinically translational between pigs and humans. Terminal contrast angiography revealed that all stimulating electrodes were retained around the splenic NVB. Confirmation of nerve function was performed at termination through eCAP recordings, assessment of physiological effects of stimulation and histological assessment of nerve health. Stimulation at termination continued to evoke an increase in mABP and decrease in SpA BF, similar to that seen on implantation. It was possible to record eCAPs distally to the implanted stimulation electrode. While not an indication of nerve function of the whole splenic NVB, successful recordings of eCAPs from a randomly selected nerve fascicle do suggest that function is maintained. Finally, histopathological analysis demonstrated that stimulation electrodes were well-tolerated with minimal or low severity changes in nerves. Changes in fascicles were restricted to Schwann cell hyperplasia and perineurial fibrosis with occasional fascicles showing evidence of intra-fascicular edema, which are unlikely to affect nerve function indicating that the system is well-tolerated.

In summary this work demonstrates that chronic conscious splenic neuromodulation can be achieved by implanting electrodes around the splenic NVB in a minimally-invasive manner and can safely deliver highly tolerable selective activation of splenic immunomodulatory circuits. Such SpN neuromodulation primes the immune system toward a pro-resolutive phenotype such that following systemic immune challenge, there is a pronounced, effective and balanced anti-inflammatory effect. This preliminary study performed exclusively in female pigs represents a significant advancement toward a better understanding of neuro-immune modulatory mechanisms in large animal species, as well as toward the development of a novel bioelectronic medicine for patients with acute or chronic inflammatory conditions targeting the near-organ autonomic nervous system of the spleen. Together these findings support the use of chronic neuromodulation of the SpN in human clinical trials to investigate the effects of SpN neuromodulation for the treatment of inflammatory disorders.

## Data Availability Statement

The original contributions presented in the study are included in the article/**Supplementary Material**. Further inquiries can be directed to the corresponding authors.

## Ethics Statement

All animal studies were ethically reviewed and carried out in accordance with Animals (Scientific Procedures) Act 1986. The protocol was approved by the Royal Veterinary College Animal Welfare and Ethical Review Board and the Galvani Bioelectronics Animal and Scientific Review Committee. All animals were transported and housed under conditions specified in the United Kingdom Animal Welfare Act 2006 and The Welfare of Farm Animals (England) Regulations 2007.

## Author Contributions 

DS, AM, and MD contributed equally to this work. DS, MD, DW, DC, and JP designed experimental plans. JP, AM, CF, and MD performed pig surgery and recovery. AM and JP were study veterinarians. DS, AM, MD, JK, IG, and KH performed pig neuromodulation and blood sampling and analysis. IG, KS, and RB refined and supported the mStim system in conjunction with Integer. ND analyzed flow cytometry data. RC and JD performed and analyzed SPM data. SO, PM, and FY designed stimulation electrode. AR analyzed histology. DS, AM, MD, JK, DC, DW, AR, JD, JP analyzed and interpreted the data. DS, AM, and MD wrote the manuscript. All authors contributed to the article and approved the submitted version.

## Conflict of Interest

Authors DS, MD, IG, SO, PM, RY, KS, RB, and DC were employed by the company Galvani Bioelectronics. Author AR was employed by the company GlaxoSmithKline. Some of the work described in this publication is the subject matter of a pending patent application. AM, CF, JK, KH, JP, RC, EG, ND, and JD declare that Galvani Bioelectronics provided funds to support their work associated with this manuscript.

The remaining authors declare that the research was conducted in the absence of any commercial or financial relationships that could be construed as a potential conflict of interest.
