# Supplementary material for: Splenic Nerve Neuromodulation Reduces Inflammation and Promotes Resolution in Chronically Implanted Pigs
Source: Front Immunol. 2021 Mar 29;12:649786. doi: 10.3389/fimmu.2021.649786 (PMC8043071; doi:10.3389/fimmu.2021.649786)
Supplement: Supplementary file 1 [file Presentation_1.pdf]

Supplementary Material

1 Supplementary Methods

1.1 Acute Terminal Study Methods

1.1.1 Refinement of SpNS Parameters – Electrophysiology

Studies were performed to refine SpNS parameters to maintain action potential stimulation in the presence of sustained neurotransmitter release (using splenic NA release as a pathway- and mechanism-relevant biomarker), whilst reducing effects on blood flow and blood pressure.

Animals were group-housed on straw bedding, given water *ad libitum* and fed a commercial pelleted sow diet. Animals were acclimatized at the research facility for a minimum of 7 days prior to the experiment; 12 h prior to the experiment food was withheld.

1.1.1.1 Surgery

Following induction of anesthesia, bilateral indwelling jugular vein catheters and one femoral arterial catheter were placed under ultrasonographic guidance. Volume-controlled mechanical ventilation was maintained for surgery and implantation. In addition, a continuous rate infusion of fentanyl (0.2 μg/kg/min) was started after induction and continued throughout the experimental procedure. Once instrumentation was complete, animals were kept on spontaneous ventilation and data collection initiated once animals were in a steady-state. Routine anesthesia monitoring included vital parameters such as electrocardiogram and invasive arterial blood pressure (systolic, diastolic and mean). Arterial blood pressure was also digitally recorded using a 16 channel PowerLab acquisition system (AD Instruments) with LabChart 8 software at 2 kHz sampling frequency.

Animals were placed in right lateral recumbency, and the left lateral abdomen was aseptically prepared and draped in a routine fashion. Using aseptic technique, a 20 cm laparotomy incision was made in the second to last intercostal space, and access to the splenic base with associated neurovasculature was aided by the use of rib retractors.

The proximal aspect of the splenic NVB was chosen as the site for neural interface implantation, as the SpA at this location was separated from the splenic vein (SpV), allowing surgical access to the NVB with minimal risk of causing iatrogenic damage to the SpN plexus. The NVB was instrumented with a bipolar circumferential cuff electrode (2.5 mm diameter, 8 mm length; cathode surface area: 0.13 cm2; #1041.2180.01, CorTec GmbH).

1.1.1.2 Recording and Analysis of Electrophysiological and Physiological Parameters

At the level of the splenic hilum, a minimum of one discrete SpN fascicle was carefully isolated and subsequently instrumented with a bipolar cuff electrode (#1041.2115.01 or #1041.2112.01; CorTec GmbH) to record evoked compound action potentials (eCAP).

Flow changes were continuously monitored via a TS420 perivascular flow module (Transonics, USA), and measurements were digitally recorded using a 16 channel PowerLab acquisition system (AD Instruments) with LabChart 8 software at 2 kHz sampling frequency.

Stimulation of the SpN plexus with various intensities, pulse durations and frequencies was delivered using a constant-current stimulator DS5 (Digitimer, UK) while eCAP and physiological responses were recorded continuously.

Electrophysiological signals were amplified and filtered (100 – 1000 Hz) using a bioamplifier (1800 2-channel microelectrode AC amplifier, A-M System) and a notch-filter (50 Hz). Nerve activity was continuously monitored using an oscilloscope, and digitally recorded with the described acquisition system with the sampling rate set at 20 kHz.

The conduction velocity of the eCAP components was calculated from the measured distance between the stimulation site and the recording site and the latency of the eCAP signal (measured from the peak of the stimulation artefact to the peak of the eCAP).

Baseline values for mABP and SpA BF were generated by averaging the measurements obtained over the last 30 s prior to each stimulation. During stimulation, values were then expressed as percentage change from this baseline value to quantify the effect of stimulation. For each parameter, the maximum change occurring during the stimulation period was used for comparison.

Least-squares regression curves were fitted for the eCAP latency and amplitude data. One-way ANOVA with multiple comparison was used to compare effects of different stimulation frequency and pattern. Statistical significance was defined as $P \leq 0.05$ and analyses were performed with commercially available statistical software (JMP Pro 13.0.0 or GraphPad Prism 8.4.2).

1.1.2 Refinement of SpNS Parameters – Noradrenaline Output

1.1.2.1 Surgery

Following cuff placement, the peritoneum was sutured above the cuff to secure it in place. Following probe placement, SpA blood flow readings were confirmed and finally the rib retractors were closed and the exposed incision covered with saline-soaked gauze sponges to protect the exposed tissue surface from dehydration.

1.1.2.2 Stimulation and Blood Sampling

Prior to stimulation, the preparation, including the smooth muscle of the vasculature was then allowed to stabilize for 30 mins. Electrical stimulation (delivered using DS5 constant current stimulator, Digitimer UK) was then applied to the SpN (12 μC, 30 Hz; biphasic, 60 s duration) as a "priming" stimulation to allow release of stored NA from the spleen that may have accumulated during the implantation procedure. This was to increase consistency in baseline samples prior to the subsequent stimulations. The preparation was then left for 60 mins.

Charges tested (0.0, 1.92, 2.88, 10.8 or 15.6 μC at 10 Hz continuous, 10.8 μC at 10 Hz burst) were selected to evaluate the efficacy profile of different stimulation paradigms, using release of NA from the spleen as a proxy.

Twenty seconds before the start of all experimental stimulations, 10 ml blood was taken over 10 s. SpNS was then delivered for 120 s. Following the initial 10 s of stimulation, blood was sampled (10 ml) every 20 s from stimulation onset to offset. A total of 7 blood samples (1 pre; 6 during) were taken for each stimulation intensity.

All blood samples were transferred to EDTA-coated tubes and then immediately centrifuged at 4 °C, 2000 xG for 5 mins. The total plasma was then collected and placed in labelled tubes and immediately frozen on dry ice. Tubes were then stored at -80 °C prior to analysis.

Frozen plasma aliquots were thawed and immediately analyzed by ELISA for quantification of NA using the Noradrenaline Sensitive ELISA (DLD Diagnostika, cat. no. ea633/96), according to manufacturer's instructions. Plates were analyzed using the Infinite® 200 PRO spectrophotometer and iControl software (Tecan Group Ltd.). NA was quantified as AUC during stimulation and for mABP and SpA BF the maximum change which occurred during stimulation was plotted. Data were assessed by one-way ANOVA using Tukey post-hoc correction for multiple comparison. Statistical significance was defined as $P \leq 0.05$ and analyses were performed with commercially available statistical software (JMP Pro 13.0.0 or GraphPad Prism 8.4.2).

1.2 Chronic Conscious Neuromodulation Study

This study developed a minimally invasive, laparoscopic technique in a translational porcine model to implant a cuff electrode around the splenic NVB and an IPG to enable delivery of chronic neuromodulation. The tolerability of SpNS in conscious, freely behaving pigs was then evaluated, prior to quantification of multiple immunological parameters in both naïve and endotoxin-challenged inflammatory conditions. These parameters included quantification of cytokines, flow cytometry and SPM analyses before, during and after LPS challenge. Under terminal anesthesia, contrast angiography, electrophysiology and histopathology demonstrated the integrity of the splenic NVB.

1.2.1 Animals

Due to the exploratory nature of the study and logistical difficulties associated with large-animal studies, animals progressed in 4 cohorts in weekly blocks. Each cohort contained animals receiving SpNS and non-stimulated sham animals to allow for any differences arising from different batches of LPS. Cohorts were used as a blocking factor in data analyses. They were individually housed in close apposition to allow visual and physical contact through slatted fencing, on straw bedding with environmental enrichment. Water was provided ad libitum and they were fed a commercial pelleted sow and weaner diet based on minimum basal and metabolic energy requirements.

1.2.2 Neuromodulation Device.

Stimulation Lead. The stimulation lead consists of a lead body with a distal end cuff electrode applied to the splenic NVB, and a proximal connector connected to the IPG manufactured from implant-grade silicone and metals. The design is able to support laparoscopic implantation for cuff placement around the splenic NVB. The cuff electrode is designed to interface with nerves located around the periphery of the splenic artery and contains two electrically active electrode arms and one inert middle arm for retention.

Implantable Pulse Generator. A commercially available implantable pulse generator (IPG; 5cc mStim IPG, Integer CCC, Uruguay; customized by Galvani Bioelectronics) was connected to the stimulation lead to electrically stimulate the SpN . Two versions of the IPG were used on the current study. In cohorts 1 and 2, the IPG was capable of stimulation up to 15 µC (15.3 mA, 980 µs pulse

width). In cohorts 3 and 4, the IPG was upgraded to provide output up to 40 µC (20.0 mA, 1980 µs). All other stimulation parameters remained identical and are detailed above. Differences in IPG output are noted in the relevant sections.

1.2.3 Implantation of the neuromodulation device

1.2.3.1 Anesthesia

Animals were started on a course (9 days) of antibiotics (amoxicillin 15 mg/kg *i.m.*) and anti-ulcer medication (omeprazole 40 mg *p.o.*) 24 h before surgery and then continued as prescribed afterwards. Food, but not water, was withheld for 18 h prior to surgery and animals received a veterinary examination to ensure health status. After induction of anesthesia as below, analgesia and anti-inflammatory meloxicam (0.4 mg/kg *i.m.*) was administered and continued in the recovery period as needed.

Animals were pre-medicated with ketamine (20 mg/kg *i.m.*) and midazolam (0.5 mg/kg *i.m.*). Fifteen min after premedication general anesthesia was induced with propofol (2 mg/kg via an auricular vein catheter). Animals were intubated with an endotracheal tube, and anesthesia was maintained with sevoflurane vaporized in a mixture of oxygen and medical air. Volume-controlled mechanical ventilation was maintained throughout surgery, as were intravenous fluids (isotonic and colloidal fluids, glucose and electrolyte supplementation as needed). Intra-operative analgesia included buprenorphine (0.02-0.04 mg/kg *i.v.*) and fentanyl (2-5 µg/kg *i.v.*). Instrumentation and monitoring included electrocardiogram (ECG), heart rate (HR), invasive arterial blood pressure (ABP; (systolic, diastolic and mean), respiratory rate (RR), pulse oximetry, capnography, spirometry, (including fraction of inspired oxygen (FiO_2), end-tidal sevoflurane (ETSev)), and core body temperature. Some of these parameters (arterial blood pressure, central venous pressure, ECG, $ETCO_2$, ETSev) were also digitally recorded using a 16 channel PowerLab acquisition system (AD Instruments) with LabChart 8 software at 2 kHz sampling frequency.

1.2.3.2 Laparoscopic surgery

A minimally-invasive laparoscopic surgical procedure was used. Animals were placed in right lateral recumbency, and the left thorax and lateral abdomen were aseptically prepared and draped in a routine fashion. A total of 7 trocars were used for each surgical procedure. The first trocar (15 mm diameter) was initially placed in the paralumbar region between the last rib and wing of the ilium using the Hasson technique. The abdomen was then insufflated with gas at a constant pressure ranging between 10-16 mmHg. At the same time the lead was prepared, inspected and tested in saline for electrical continuity (impedance). The laparoscopic camera was then inserted and used to select the ideal location for successive trocars. A second trocar (5 mm) was then placed along the cranio-ventral axis of the abdomen to provide an access port for stomach retraction tools. A third trocar (12 mm) was placed approximately 20 cm caudal from the second trocar for spleen retraction. Three additional trocars (12 mm) were placed approximately 15-20 cm dorsal from the retraction trocars to form a triangle just ventral to the last and second to last ribs. The two trocars at the base of the triangle (n.4 and 5) were used for dissection and cuff implantation, while the other (n.6) was used for the laparoscope.

The head of the spleen was retracted caudally by fixing a self-retaining laparoscopic Overholt clamp at the base of the renosplenic ligament. The stomach was then retracted ventrally by using an articulating cobra liver retractor to provide access to the splenic NVB. Initial dissection of the peritoneum overlying the distal (closer to the spleen) splenic NVB was performed using a harmonic

laparoscopic scalpel to prevent bleeding. The splenic NVB was then isolated by blunt dissection using a Maryland tool, 60 and 90-degree Overholt dissecting instruments and scissors. A region of approximately 2 cm of NVB was freed from the connective tissue and separated from the splenic vein. The stimulation lead was then prepared for deployment into the abdomen. A seventh trocar (5 mm) (n.7) was placed between the two access ports used for dissection. The lead was then introduced into the abdomen using atraumatic graspers to manipulate the lead. The lead was exteriorized by pulling the lead cap through trocar n.7. The circumferential cuff electrode was then implanted around the splenic NVB and impedance was checked using a Minirator (MR-PRO, NTI Audio, Switzerland) to confirm electrical continuity.

Stimulation was then performed with an external pulse generator (EPG; DS5, Digitimer, UK) to confirm electrical integrity and physiological functionality by a measured increase in mABP. Intraoperative splenic nerve neuromodulation (10 Hz continuous; 60 s) consistently induces changes in physiological biomarkers [4] which enabled robust confirmation of nerve-target activation via the test system. Specifically, systolic, diastolic and mean arterial blood pressure (mABP) are increased during splenic nerve stimulation under anesthesia; these are caused by smooth muscle contraction within the artery and spleen. These changes are directly correlated with the amplitude and frequency of stimulation, and such changes are resolved upon the cessation of stimulation.

A 3 min stable period of no stimulation was performed to obtain baseline values, including mABP. Stimulation (see **Table S5** for stimulation parameters) was applied via the IPG; the ability to evoke an increase in mABP was used to demonstrate function of the implanted lead and effects on target physiology. A period of no stimulation (minimum 120 s) was performed to allow recovery of cardiovascular parameters (mABP) to ± 10% of pre-stimulation values between each stimulation.

A subcutaneous pocket was created to accommodate the implantable pulse generator (IPG; Integer, CCC, Uruguay) dorsally, approximately above the third to last rib and in line with the position of the n.7 trocar. An incision of 5-8 cm was performed and pocket created by blunt dissection between subcutaneous fat and muscle layer. The n.7 trocar was carefully removed, and the lead tunneled subcutaneously to the IPG pocket on the lateral thorax.

Subsequently, the lead connector was attached to the IPG and the IPG implanted into the subcutaneous pocket on the lateral thorax. Instruments and trocars were removed. A series of stimulations were then applied via the IPG to confirm functionality by comparison to changes evoked with the EPG, and IPG communication and charging were confirmed. The trocar locations were then sutured closed. Stimulation was then delivered via the IPG up to either 15 or 40 μC using a 10 Hz continuous paradigm as detailed in main methods.

1.2.3.3 Vascular access port (VAP) implantation

Following implantation and stimulation, animals were placed in dorsal recumbency and the neck and scapula region prepared and draped for aseptic surgery. An intravenous catheter was placed, as below, in the left external jugular vein using a minimally invasive ultrasound-guided approach and terminated with a subcutaneous vascular access port (Le Grand CompanionPort (CP305K) – Norfolk Vet Products, Skokie, IL, USA). The VAP consisted of a titanium port with a silicone septum and an attachable rounded tip silicone catheter (7 French). Both the port and catheter were flushed with 0.9% saline prior to insertion.

A 1 cm incision was made in the skin overlying the left external jugular vein in the mid-cervical region. An 8 Fr catheter introducer was placed into the jugular vein under ultrasound guidance and the catheter for the VAP passed through this introducer and advanced 9-10 cm distally. Tip localization was confirmed using fluoroscopy to ensure location was proximal to the heart. For placement of the port, a 5- to 6-cm curvilinear incision was made dorso-cranial to the scapula and 5 cm lateral to midline dorsal neck. Subcutaneous tissues overlying trapezius muscle were undermined to create a pocket for the port. The catheter was tunneled dorsally between the skin and subcutaneous tissues and attached to the port. The port was secured to the underlying musculature by using 3-0 polydioxanone suture at 2 anchor points on the port. Catheter patency was confirmed intraoperatively through withdrawal of a blood sample. The port and catheter were flushed with 5 to 6 mL 0.9% saline and locked with 5 mL heparinized saline (500 IU/mL, Hospira). VAPs were maintained as per below until blood sampling.

1.2.3.4 VAP maintenance and use

VAPs were maintained in all animals during the study period; they were accessed either for experimental procedures or minimally every 2 weeks for maintenance. Briefly, the animal was restrained in a crate to which it was habituated, and aseptic technique used throughout. Topical local anesthetic cream (lignocaine 2.5% and prilocaine 2.5%; EMLA 5% cream; Aspen medical) was rubbed in the skin over the access port, palpable under the skin, and left for a minimum of 1 h. After final skin preparation, the port was located and stabilized by holding the edge and a right angled Huber needle (22G 1"; Norfolk Vet Products, Skokie, IL, USA), attached to an extension set and syringe containing 0.9% saline flush solution, was inserted through the skin and into the dome of the port. Flush solution (5 mL) was introduced into the catheter, and then a syringe used to withdraw at least 3 times the volume of the VAP system, including the flush, which was discarded. Following this, either blood samples were taken or LPS injected. The catheter was then flushed again with 0.9% saline (3 times volume VAP system), followed by lock solution. If further samples were to be taken within 24 h, the needle remained indwelling in the port, attached to a sealed extension set and secured around the animal. When the system was accessed within 24 h and the needle left in place, it was flushed and locked with 5 mL heparinized saline (100 U/mL); when samples were not taken within that time prior to removing the needle, a lock solution of 5 mL heparinized saline (500 U/mL) was placed in the VAP.

1.2.4 Effects of Chronic SpN Neuromodulation in Naïve Animals

1.2.4.1 Tolerability to SpNS as determined by behavioral responses

After a 14-day recovery period from surgery, in those pigs assigned to the neuromodulation group, tolerability to SpNS (as per the conscious stimulation paradigm; **table S5**), was determined. Impedance was measured via the IPG before, during and after each stimulation session. Animals in the sham group did not receive any neuromodulation. Behavioral responses indicative of perception to SpNS were scored by two independent observers. Responses identified as most severe: abdominal wall contractions, distressed vocalization and excessive agitation/psychomotor activity, warranted immediate cessation of stimulation if seen individually and only on a single occasion. The animal was allowed to recover for five minutes, and following veterinary assessment, stimulated again at the next lowest intensity before attempt at step-up made again. Other less severe behaviors (startle response, scratching/rubbing, nose bumping against a solid object, kneeling, squatting, stomping and stretching) were scored as absent or present; and if present whether as a single occasion, intermittent or continuous. If a combination of two or more these behaviors was seen at greater than a single frequency, stimulation was ceased and after a five-minute recovery, retested at the same intensity. If the response was observed during the second stimulation at the same level, no further increase in stimulation would occur and

effects of stimulation reassessed at the next lowest intensity to confirm absence of limiting behavioral changes. If no response was observed during the second stimulation at the same level, the stimulation would be increased in 1 mA levels until either an observed response or IPG maximum was reached.

1.2.4.2 Blood Sampling at Baseline in Naïve Animals

Animals were then left for a further 14 days with no stimulation. Blood samples were then taken from all animals for baseline blood testing at Days -2, -1 and 0 and then following initiation of SpNS, on Days 2 and 7 from the VAP (as per above protocol). Bloods for *ex vivo* LPS cytokine assay and flow cytometry were collected in sodium heparin-coated tubes. Bloods for clinical biochemistry were collected in lithium heparin- and non-coated tubes. Blood for hematology was collected in EDTA-coated tubes.

1.2.4.3 LPS preparation methods

A 1 mg vial of LPS (Purified lipopolysaccharides from the cell membrane of *Escherichia coli* O111:B4; Sigma Aldrich) was reconstituted with 1 mL sterile saline to give 1 mg/mL solution. The vial was vortexed for 20 s and then sonicated for 5 min. Stock solution aliquots of 500 µg/mL were then made by adding 100 µL of 1 mg/mL LPS to 100 µL saline in individual tubes. These were again vortexed for 20 s and sonicated for 5 min and stored for up to 12 h at 4°C. Within 20 min of use, working solutions of LPS were made (50, 5 or 0 µg/mL): tubes were again vortexed and sonicated as above, before serial dilutions with sterile saline. Vortex and sonication were done between each dilution step. Working solutions were sonicated again immediately prior to incubation with blood samples.

1.2.4.4 Ex vivo LPS Cytokine Assay

Within 15 min of blood collection, blood tubes were inverted to resuspend cells and 20 µL of working LPS dilutions (50, 5 or 0 µg/mL) were added to culture tubes, followed by addition of 980 µL of blood to each tube to achieve final concentrations of 1000, 100 or 0 ng/mL LPS. Two replicates were performed per final concentration. Samples were mixed by 3 inversions and transferred to a 37°C incubator, flat on a rocker for 4 h. Plasma was then separated by centrifugation for 10 min at 2000 xG to pellet cells and then removed by pipette and stored in cryovials at -80°C.

Samples were thawed and TNF-α analyzed by commercially available ELISA kits (Porcine TNF-α; DY690B; DuoSet Solid Phase Sandwich ELISA, R&D Systems), run as per the manufacturer's instruction. All samples were run as technical replicates (n=3) for each time-point and LPS concentration.

1.2.4.5 Flow cytometry methods

Data recorded by the flow cytometer was analyzed using FlowJo™ software (version 10.6.2). Each blood sample was divided in 7 aliquots to generate 7 different panels: 1) unstained; 2) isotype control for panel 3; 3) antibodies against CD4 and CD8; 4) isotype control for panel 5; 5) antibodies against CD14/CD16; 6) isotype control for panel 7; 7) antibodies against CD172a/CD163. Gates were applied according to the gating strategy (**fig. S4).** Identical gates were applied for all samples (all experimental animals, days and cohorts). Panel 3 was used to distinguish T cell subsets based on their CD4 and CD8 expression; Panels 5 and 7 were used to analyze the monocyte population. Simple gating on all $CD14^+$, $CD16^+$ or $CD172^+$ cells resulted in inclusion of granulocyte and lymphocyte populations (**fig. S4**). Because our primary interest in Panels 5 and 7 was in the monocyte population, gating was done in a view where the marker was plotted against Side Scatter. The position of monocyte population on the

Side Scatter axis was, as expected, between lymphocytes and granulocytes. Although there is a slight overlap with these populations, using side scatter in combination with the marker resulted in a reliable separation of these three subsets. This strategy was therefore used as the final gating strategy. Results are expressed as a percentage of its parent population. In the case of CD14 expression on $CD16^{+}$ monocytes, median fluorescence intensity was measured along with percentage of $CD14^{low}$ and $CD14^{high}$ cells.

1.2.5 Effects of Chronic SpN Neuromodulation During Endotoxemia

1.2.5.1 LPS preparation for in vivo experiments

LPS stock solution of 1 mg/mL was prepared as above. A 1:10 dilution of this was made by diluting 100 µL of stock solution into 900 µL sterile saline, which was then vortexed for 20 s. The final concentration of LPS to be given based on the animal's body weight was calculated (0.025 µg/kg) and a 1 mL solution of 2x final LPS concentration solution made into a glass vial, vortexed for 20 s and kept on ice until use (within 30 min). Just prior to use, the vial was sonicated for 5 min, 500 µL were then diluted in 9.5 mL sterile saline and the solution injected IV over 2 min into each animal.

1.2.5.2 In vivo LPS and cytokines assays

Immediately prior to LPS, and following LPS injection, blood samples were drawn in the following tubes: flow cytometry in sodium heparin-coated; cytokine analysis, SPM analysis and hematology in EDTA-coated; clinical biochemistry in lithium heparin and non-coated.

Bloods for cytokine analysis, hematology and clinical biochemistry were drawn at: 0 (baseline), 0.5, 1, 1.5, 2, 3, 4, 6, 8 and 24 h. Bloods for flow cytometry were drawn at: 0 (baseline), 3 and 24 h. Bloods for SPM analysis were drawn at: 0 (baseline), 0.5, 3 and 24 h.

1.2.5.3 Monitoring of LPS-Induced Clinical Reactions

Clinical reactions to LPS were carefully monitored to ensure (as predicted from the preliminary study) that only mild to moderate clinical signs occurred, such as shivering and lethargy; animals were predicted to remain responsive and appetent throughout.

1.2.5.4 Targeted lipid mediator profiling

Prior to sample extraction, deuterated internal standards, representing each region in the chromatographic analysis were added to facilitate quantification. Samples were kept at -20° C for a minimum of 45 mins to allow protein precipitation. Supernatants were subjected to solid phase extraction, methyl formate and methanol fraction collected, dried and suspended in phase (methanol/water, 1:1, vol/vol) for injection on a Shimadzu LC-20AD HPLC and a Shimadzu SIL-20AC autoinjector, paired with a QTrap 6500+ (Sciex). An Agilent Poroshell 120 EC-C18 column (100 mm x 4.6 mm x 2.7 µm) was kept at 50° C and mediators eluted using a mobile phase consisting of methanol/water/acetic acid. QTrap 6500+ was operated using a multiple reaction monitoring method (see Dalli, J., et al., *Lipid Mediator Metabolomics Via LC-MS/MS Profiling and Analysis.* Methods Mol Biol, 2018. **1730**: p. 59-72). Each lipid mediator was identified using established criteria including matching retention time to synthetic or authentic standards, an AUC >2000 counts and matching of at least 6 diagnostic ions in the MS/MS. Calibration curves were obtained for each mediator using lipid mediator mixtures at 0.78, 1.56, 3.12, 6.25, 12.5, 25, 50, 100, and 200 pg that gave linear calibration curves with an r2 values of 0.98– 0.99.

1.3 Terminally-Anesthetized Procedures

Anesthesia was performed using the same induction protocol as described previously for the chronic neuromodulation study.

1.3.1 Contrast Angiography

Contrast angiography was performed in an aseptic CT procedure room. Animals were positioned in dorsal recumbency and maintained under anesthesia (as described above). The skin over the right femoral artery was prepared in an aseptic manner and sterility maintained throughout the imaging procedure. An introducer was placed in the femoral artery under ultrasound guidance and secured for the duration of the imaging procedure. A catheter (6 or 7 Fr Avanti$^+$ Catheter; Cordis, Florida, USA) was placed through the introducer and advanced to the celiac artery for contrast angiography. Contrast agent (Iohexol, Omnipaque™ 300 mgI/mL; GE Healthcare, Amersham, UK) was injected through the catheter into the celiac artery to obtain scans from the dorsal, ventral, lateral, and oblique views/angles. Imaging was performed at a minimum along the long axis of the SpA. Upon completion of angiography, the intravascular catheter and introducer were removed and pressure applied to ensure hemostasis.

1.3.2 Histopathology

Five minutes prior to euthanasia 5000 IU heparin IV was administered to prevent post-mortem clotting.

Following gross pathology examination, tissues were harvested as follows:

- The entire segment of splenic artery including the cuff and surrounding muscle/fat tissue. The entire block of tissue was attached to a cork board to maintain orientation during fixation and the proximal and distal ends labelled. The cuff was left *in situ*.
- Tissues from lead and IPG regions: One section of pancreas closest to the neural interface (NI) site, one section proximal (by approx. 5 cm) and one section distal (by approx. 5cm).
- One section of spleen from an area close to the NI implantation site and one section lateral to the first section (by approx. 5 cm).
- One section of liver from the left lateral lobe, in the immediate vicinity of the implant site.

All tissues were immersed in 10% neutral buffered formalin solution (minimum 10x volume of tissue sample) for 48 hours prior to immersion in 70% alcohol.

1.3.3 Histology

For chronically implanted and surgical sham animals, four tissue blocks (fixed tissue) were prepared. Following fixation, the proximal and distal margins of the neural interface were marked with tissue marking ink and/or sutures and the cuff was carefully removed. The implant region was trimmed to generate four tissue blocks (P, MA, MB and D, as indicated in **fig. S14**). Three to four sequential 4-5 μm thick sections were taken from each block at the approximate location of the blue dashed arrows. One section at each level was stained with hematoxylin and eosin (H&E) and one with Gomori's elastin trichrome.

Sections of the splenic artery were assessed for microscopic changes including, but not limited to, degeneration of nerves, inflammatory infiltrates, necrosis, fibrosis, hemorrhage, neo-intimal hyperplasia/stenosis, and medial degeneration. Three H&E-stained tissue sections of the spleen and

pancreas in the vicinity of the neural interface and 5 cm proximal and distal to the implant site and one section of the liver were also examined microscopically.

All changes were graded for severity using a semi-quantitative four or five-point grading scheme, based on the relative extent of each lesion within a section. Grading criteria for changes to nerve fascicles and surrounding connective tissue are listed in **table S7**. All tissue sections were examined microscopically by a board-certified veterinary pathologist.

2 Supplementary Figures

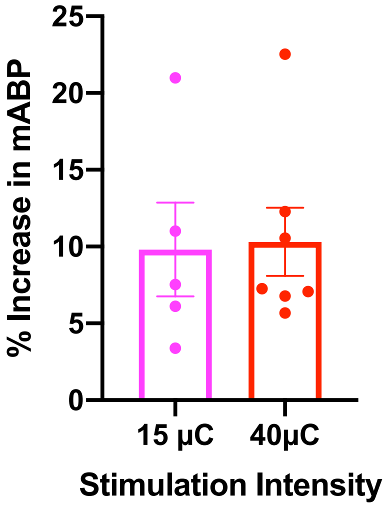

Figure S1. Continuous Intraoperative Stimulation of the Splenic NVB Confirms Target Engagement and Functionality of the Implanted Neuromodulation System. The SpNVB was stimulated at either 15 µC (15.3 mA; 980 µs pulse width; n=5) or 40 µC (20.0 mA; 1980 µs pulse width; n=7) using a 10 Hz continuous paradigm for 60 s. Graph showing that stimulation evoked an increase in mABP with no difference between groups. Data are presented as mean ± s.e.m.

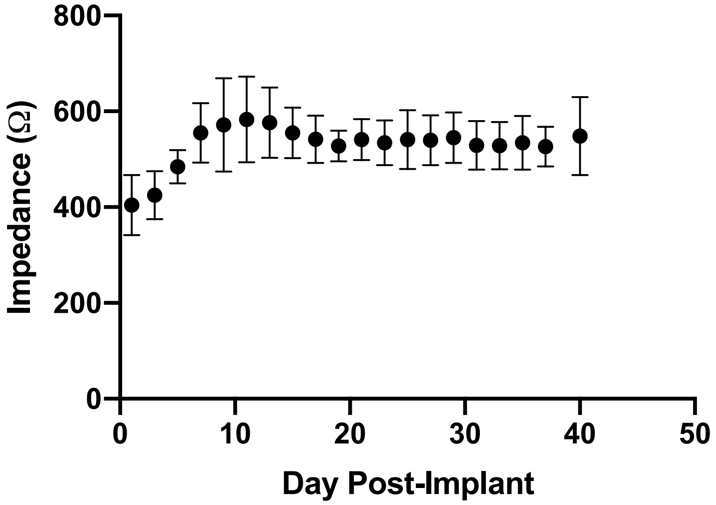

Figure S2. Serial Impedance Measurements Confirm Continued Electrode Integrity. Graph showing that following implantation, impedance was 404.33±62.68 Ω, and peaked around 11 days post-implantation (583.21±89.78 Ω) before plateauing around 540 Ω for the remainder of the study (n=10 of 12 animals). Data are presented as mean ± s.e.m..

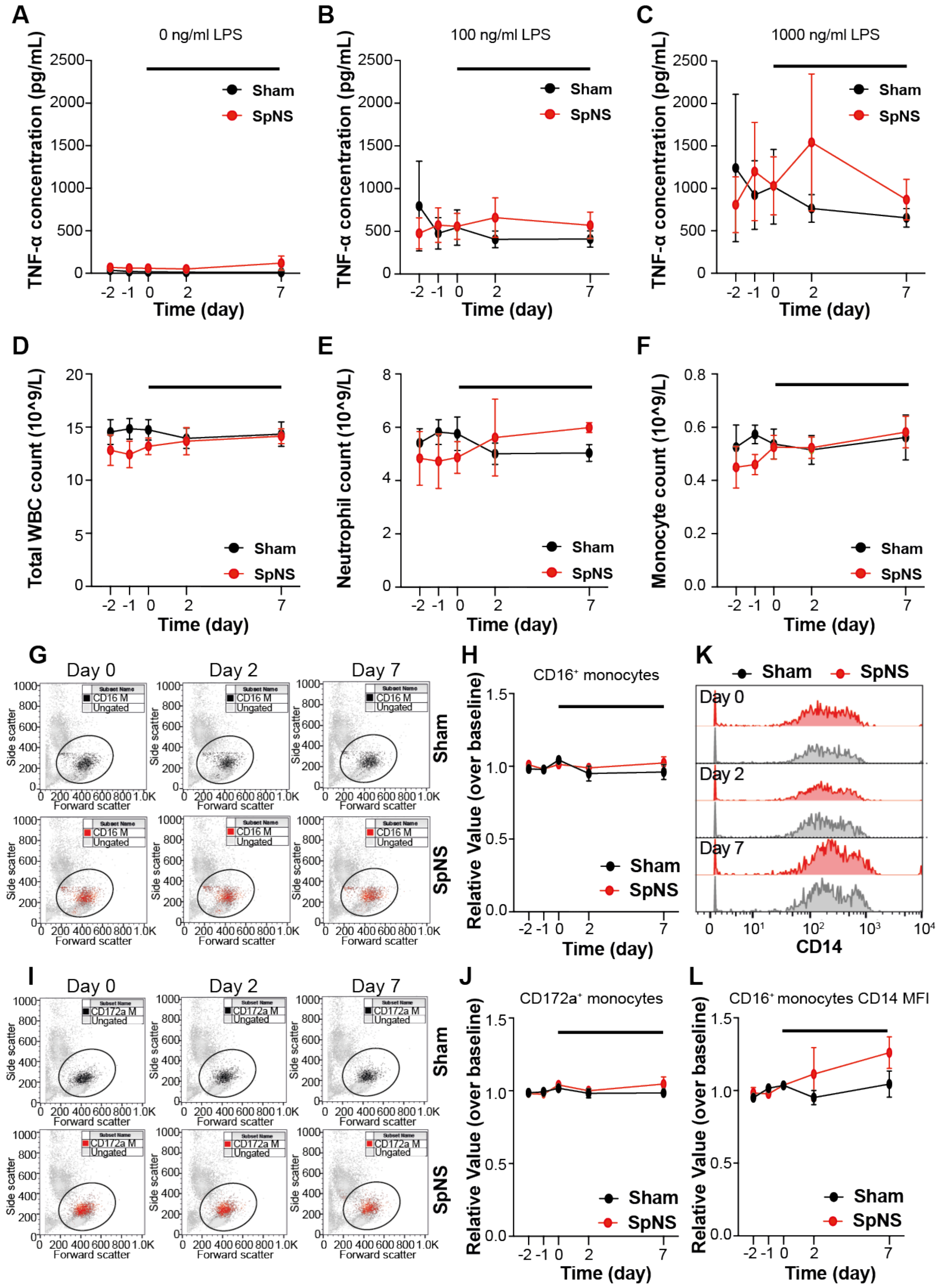
A
0 ng/ml LPS
B
100 ng/ml LPS
C
1000 ng/ml LPS
TNF-α concentration (pg/mL)
Time (day)
Sham
SpNS
D
Total WBC count (10^9/L)
E
Neutrophil count (10^9/L)
F
Monocyte count (10^9/L)
G
Day 0
Day 2
Day 7
Side scatter
Forward scatter
CD16 M
Ungated
H
CD16⁺ monocytes
Relative Value (over baseline)
K
CD14
I
CD172a M
J
CD172a⁺ monocytes
L
CD16⁺ monocytes CD14 MFI

Figure S3. Chronic SpN Neuromodulation Does Not Cause Systemic Immune Suppression in Naïve Pigs. (**A**-**C**) Graphs showing the plasma TNF-α concentration following incubation of peripheral whole blood with either 0, 100 or 1000 ng/mL of LPS, performed at the different time points. (**D**-**F**) Graphs showing total white blood cell (**D**), neutrophil (**E**) or monocyte (**F**) cell counts from peripheral blood over time. (**G** and **I**) Dot plots (forward scatter vs. side scatter view) showing the changes over time (0, 2 and 7 days) of $CD16^+$ (**G**) and $CD172a^+$ (**I**) gated monocytes population in a sham (black) and a SpNS (red) representative animals. (**H** and **J**) Graphs showing quantification of peripheral blood monocytes stained with antibodies against CD16 (**H**) or CD172a (**J**) over time. (**K**) Representative histograms showing the changes over time of CD14 expression on $CD16^+$ monocytes in a sham and a SpNS representative animals. (**L**) Quantification of the median fluorescence intensity (MFI) of CD14 expression on $CD16^+$ monocytes. (**H**, **J**, **L**) Data are expressed as relative change over the average baseline value (average between -2, -1 and 0). (**A**-**F** and **H, J, L**) Data are expressed as mean ± s.e.m.. The sham group is shown in black and SpNS group in red. (**A**-**F**, **H**, **J** and **L**) The black bar indicates the SpNS period. (**A-F**) n=6 for SpNS; n=6 for sham. (**H, J, L**) n=3 for SpNS; n=4 for sham.

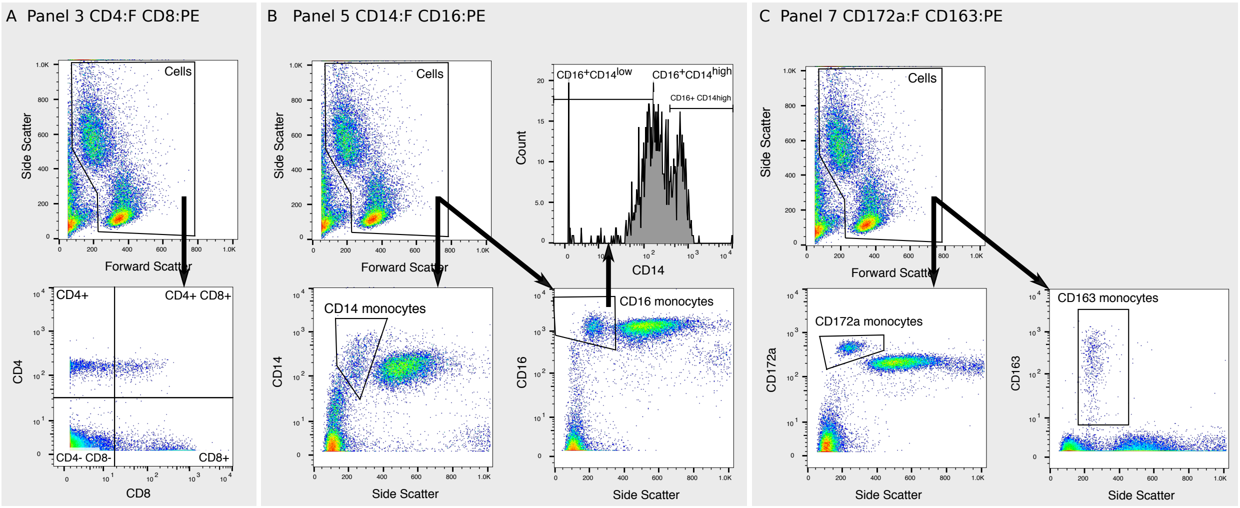

Figure S4. Gating Strategy for Flow Cytometric Analysis. (A) Gating strategy for Panel 3: Initial gate on all cells in FSC vs. SSC view, then quadrant division of the CD8 vs. CD4 view, to identify $CD4^+$, $CD8^+$ and $CD4^+CD8^+$ (double positive) populations. **(B)** Gating strategy for Panel 5: Initial gate on all cells in FSC vs. SSC view, then gate on $CD14^+$ monocytes in SSC vs. CD14 view, or gate on $CD16^+$ monocytes in SSC vs. CD16 view. $CD16^+$ monocytes were further analyzed for their CD14 expression and split into $CD14^{low}$ and $CD14^{high}$ populations. **(C)** Gating strategy for Panel 7: Initial gate on all cells in FSC vs. SSC view, then gate on $CD172a^+$ monocytes in SSC vs. CD172a view, or gate on $CD163^+$ monocytes in SSC vs. CD163 view. FSC = Forward Scatter, SSC = Side Scatter.

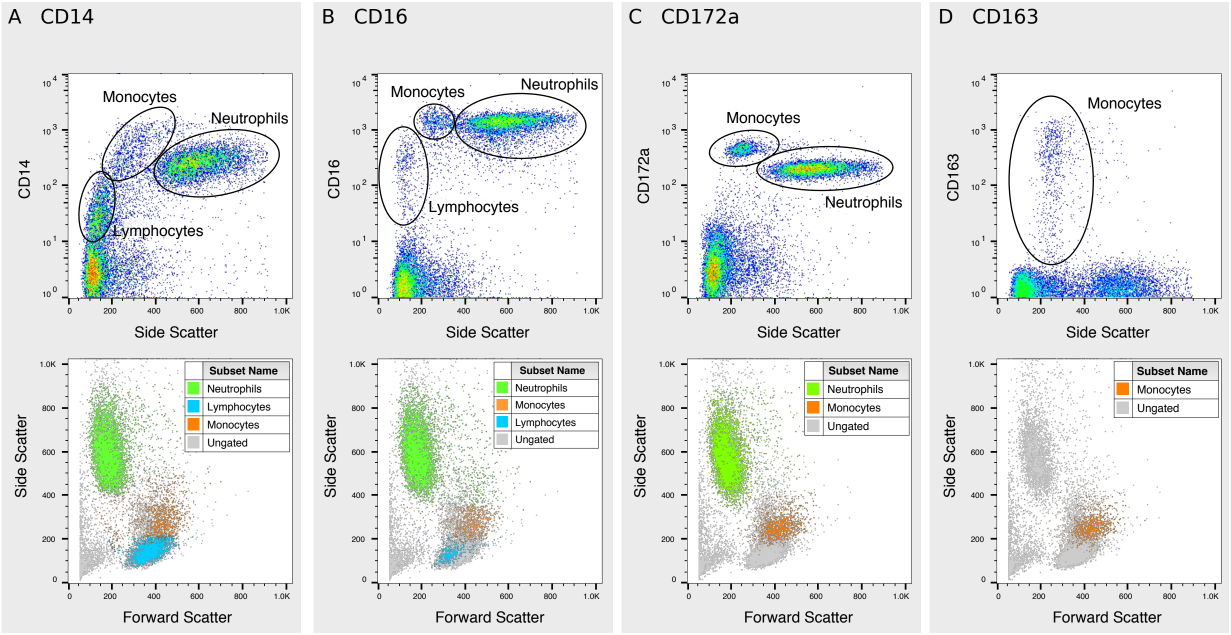

Figure S5. Exploration of the Marker Expression on Populations of Peripheral Blood Monocytes. On the bottom set of plots (FSC vs. SSC view), blue cells mapped into the expected lymphocyte region, orange into monocyte region and green into granulocyte region. **(A)** $CD14^+$ cells were found in all three mononuclear cell subsets (low expression in a subset of lymphocytes, high expression in monocytes and granulocytes). **(B)** $CD16^+$ cells were found in all three mononuclear cell subsets (low expression in lymphocytes, high expression in monocytes and granulocytes). **(C)** $CD172a^+$ cells were found in monocyte and granulocyte populations (high expression in both). **(D)** $CD163^+$ cells were only found in the monocyte population. For all four markers, the monocyte population could be reliably separated on the SSC vs. marker view.

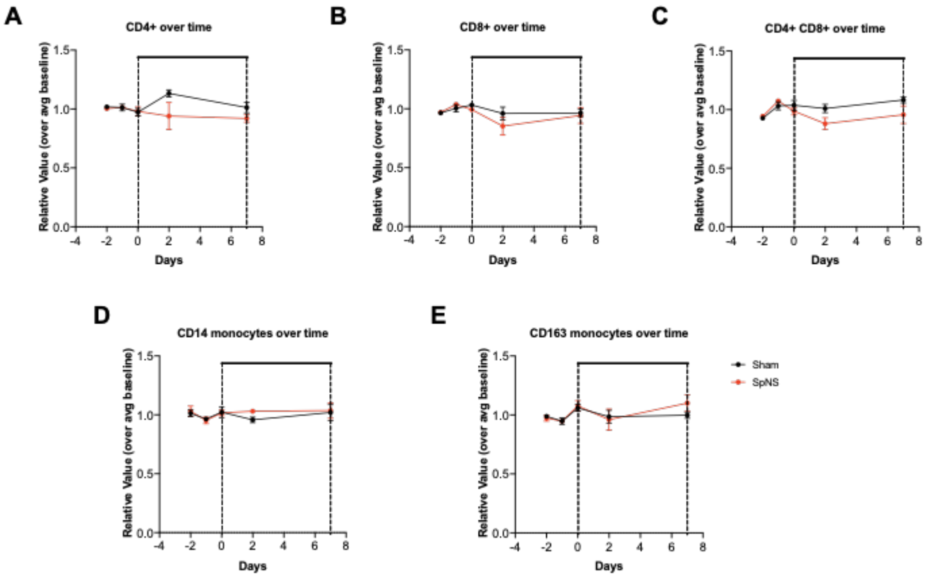

Figure S6. Chronic SpN Neuromodulation Does Not Affect Peripheral Leukocytes in Naïve Pigs. Graphs showing the quantification of peripheral leukocytes. In particular the changes in peripheral CD4+ (**A**), CD8+ (**B**), CD4+CD8+ (**C**) lymphocytes, and CD14+ (**D**), or CD163+ (**E**) monocytes are shown. Data were expressed as relative change of the proportion of positive cells over the baseline (average value of -2, -1 and 0 time point) at the different time points. Data are shown as mean ± s.e.m. The gating strategy in shown in fig. S4 and S5. (**A-E)** n=3 for SpNS; n=4 for sham. The sham group is shown in black and SpNS group in red.

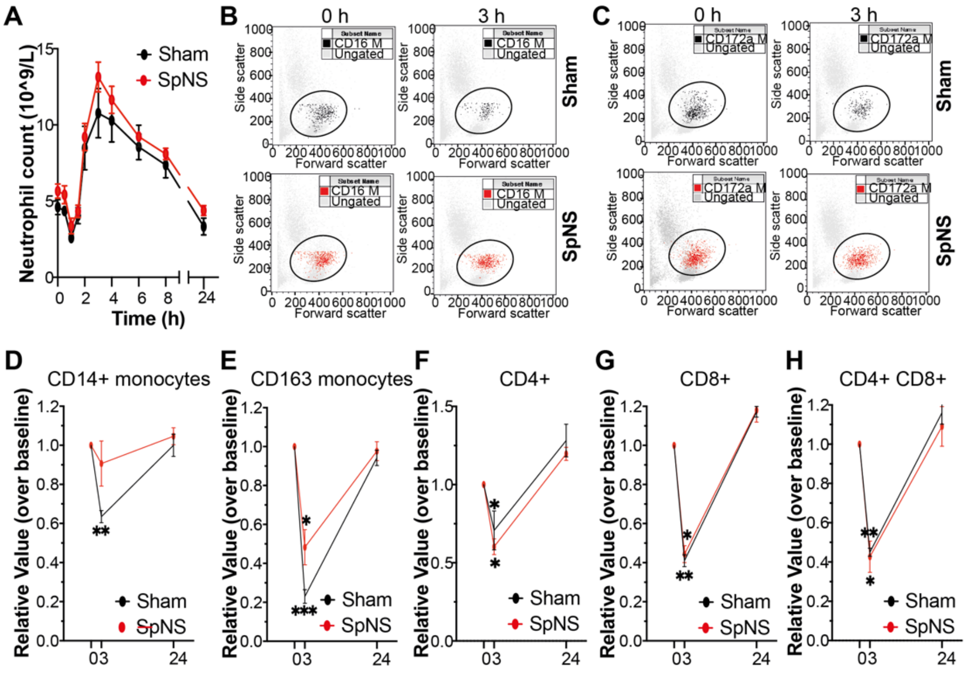

Figure S7. Chronic SpN Neuromodulation Affects Peripheral Leukocytes During Endotoxemia. Graphs showing the quantification of peripheral leukocytes. In particular the changes in peripheral neutrophil (**B**); CD14+ (**D**) or CD163+ (**E**) monocytes; CD4+ (**F**), CD8+ (**G**) or CD4+CD8+ (**H**) lymphocytes. Dot plots (forward scatter vs. side scatter view) showing the changes over time (0, 3 and 24 hrs post LPS) of CD16$^+$ (**B**) and CD172a$^+$ (**C**) gated monocyte populations in a sham and a SpNS representative pig. (**D-H**) data were expressed as relative change of the proportion of positive cells over the baseline (value at time 0 h, prior to LPS injection) at the different time points. Data are shown as mean ± s.e.m.. The gating strategy in shown in fig. S6 and S7. n=3 for SpNS; n=4 for sham. The sham group is shown in black and SpNS group in red. *, P≤0.05; **, P≤0.005; ***, P≤0.001 *vs* baseline. (**A**) n=6 for SpNS; n=5 for sham.

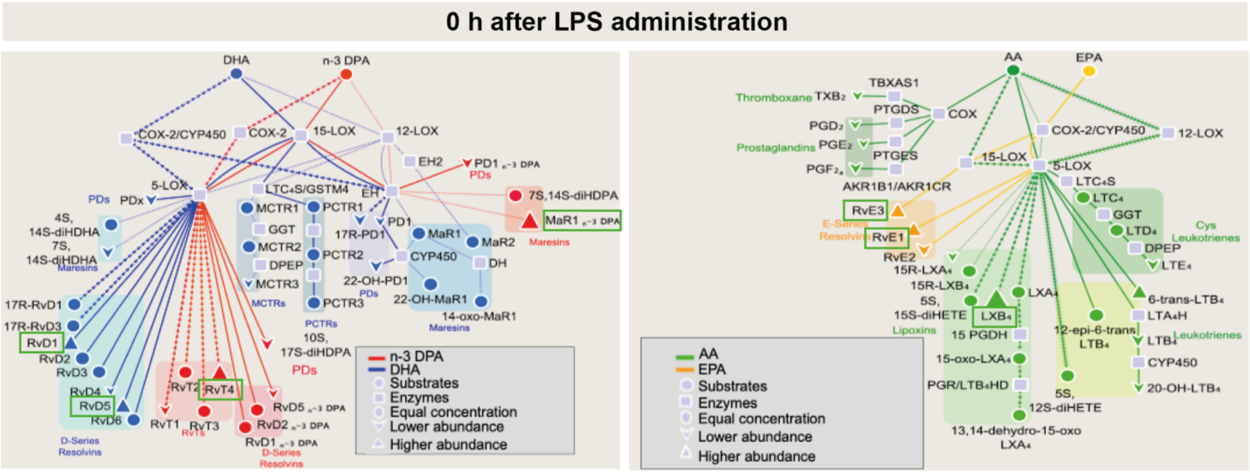

Figure S8. Chronic SpN Neuromodulation Leads to Long Term Reprograming of Peripheral Blood Lipid Mediator Profiles in Response to LPS. Porcine splenic nerve was stimulated chronically for 8 days prior to systemic LPS administration and blood was collected just prior to LPS administration (0 h). Plasma lipid mediators were investigated using LC-MS/MS-based profiling. Flux down each of the bioactive metabolomes was assessed. Pathway analysis for the differential expression of mediators from the (top panel) EPA and AA, and (bottom panel) DHA and n-3 DPA bioactive metabolomes in SpNS group when compared to Sham group. Results are expressed as the fold change. n=5 for Sham group and n=6 for SpNS group.

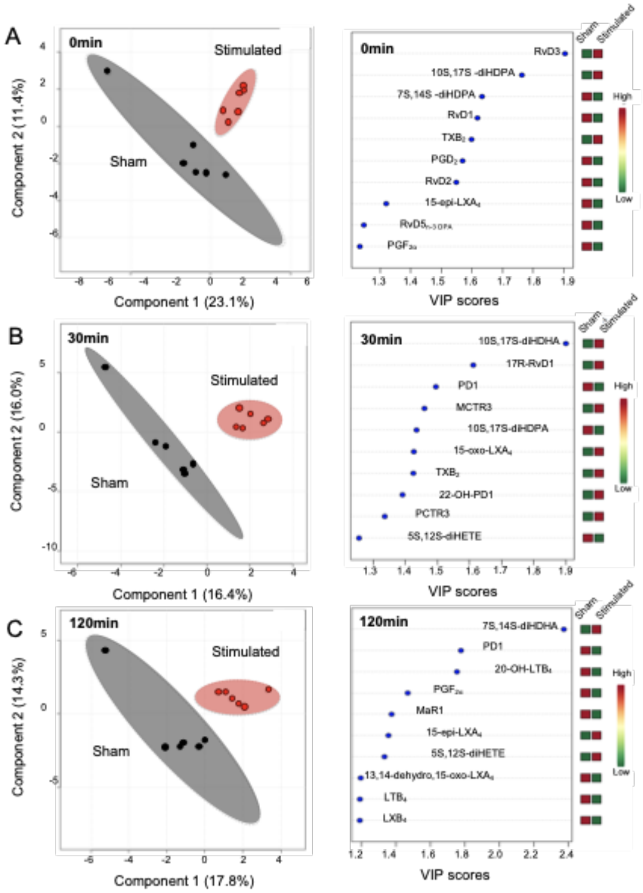

Figure S9. Differential Regulation of Plasma Lipid Mediator Profiles Following Acute SpN Neuromodulation. Terminally anesthetized pigs received sham or SpN stimulation for 3 h (from –2 h to +1 h relative to LPS administration). Plasma was collected (A) at 0 h (immediately prior to LPS challenge), (B) 0.5 h, and (C) 2 h post LPS challenge and LM were investigated using LC-MS/MS-based lipid mediator profiling. Results were interrogated using Partial Least Square Discriminant Analysis. (Left panels) Display Score Plots. Colored area represents the 95% interval confidence. (Right panels) Plots displaying the LM with the 10 highest VIP scores from component 1. n=6 for Sham group and n=6 for SpNS group.

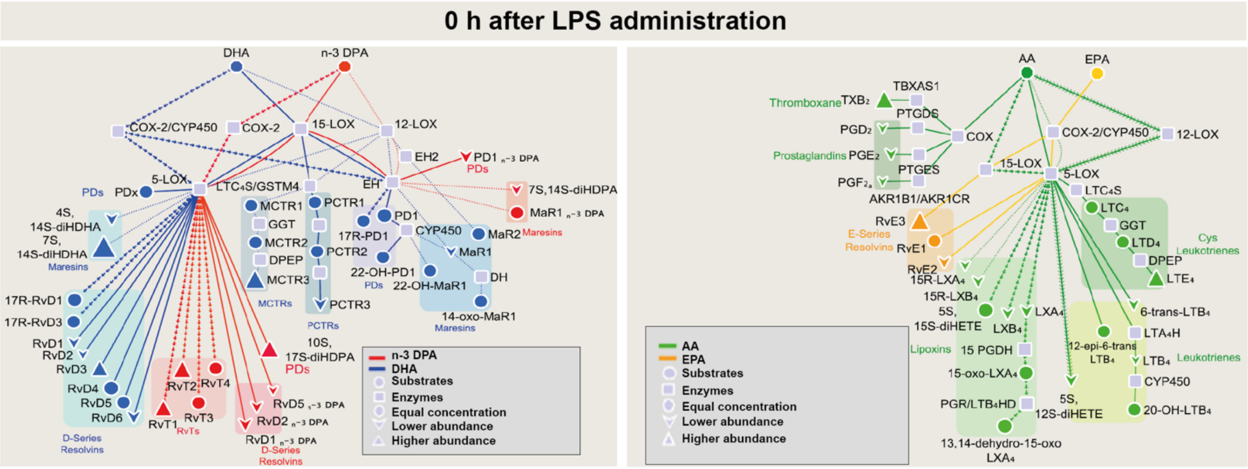

Figure S10. Acute SpN Neuromodulation Downregulates Plasma Prostaglandins and Upregulates SPM. Terminally anesthetized pigs received sham or SpN stimulation for 2 h prior to LPS challenge, blood was collected just prior to LPS injection and LM were investigated using LC-MS/MS-based lipid mediator profiling. Flux down each of the bioactive metabolomes was assessed. Pathway analysis for the differential expression of mediators from the (left panel) EPA and AA, and (right panel) DHA and n-3 DPA bioactive metabolomes in Stimulated group when compared to Sham group. Results are expressed as the fold change. n=6 for Sham group and n=6 for SpNS group.

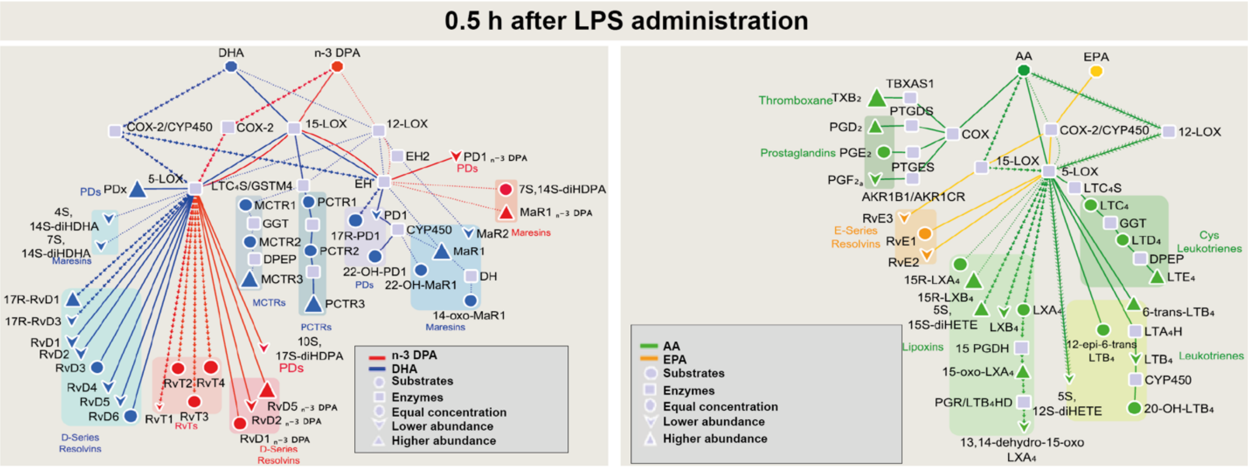

Figure S11. Acute SpN Neuromodulation Shifts Plasma Lipid Mediator Profiles in Response To LPS. Terminally anesthetized pigs received sham or SpN stimulation for 3 h (from –2 h to +1 h relative to LPS administration). Pigs were challenged with LPS at time 0 h, blood was collected after 0.5 h and LM were investigated using LC-MS/MS-based lipid mediator profiling. Flux down each of the bioactive metabolomes was assessed. Pathway analysis for the differential expression of mediators from the (left panel) EPA and AA, and (right panel) DHA and n-3 DPA bioactive metabolomes in Stimulated group when compared to Sham group. Results are expressed as the fold change. n=6 for Sham group and n=6 for SpNS group.

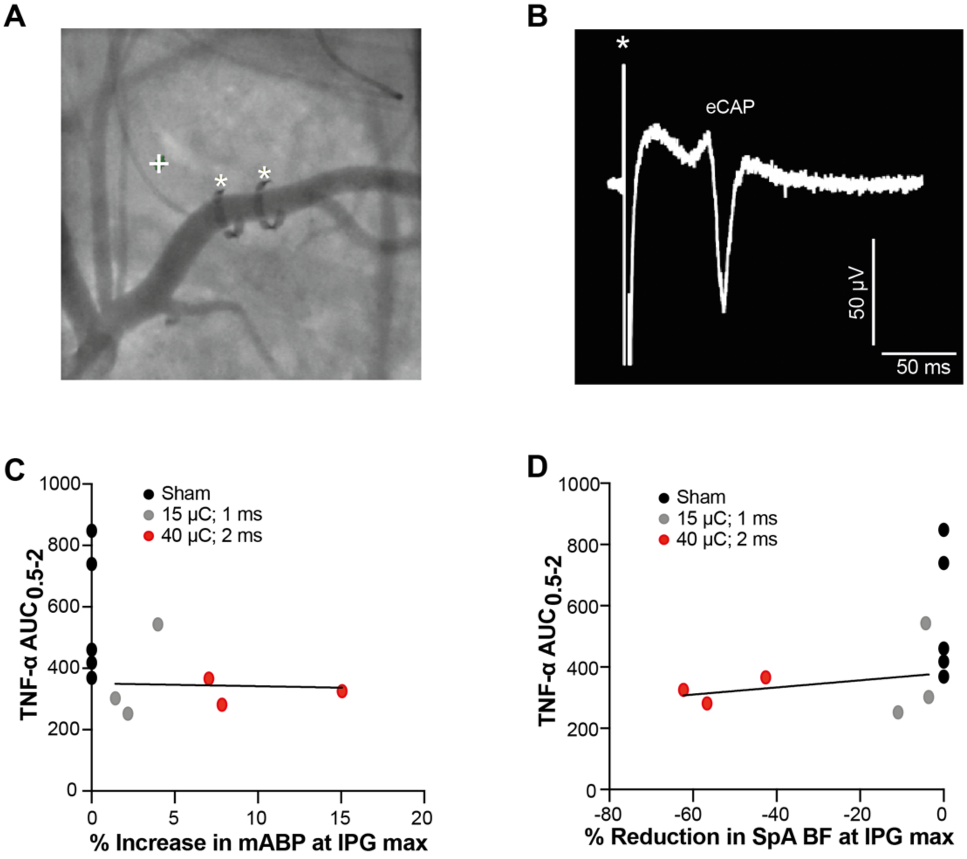

Figure S12. CT Angiogram, Electrophysiological and Physiological Assessment Confirms Integrity of Electrode-Nerve-Spleen Circuitry at Termination. (**A**) Representative contrast CT angiogram showing retention of stimulation lead cuff around the SpA. Injection of contrast medium into the celiac artery allowed visualization of the SpA. Also shown are the stimulation lead electrodes (*) and stimulation lead (+). (**B**) Representative image of a SpN eCAP recorded distally with respect to the stimulating cuff. The eCAP is the average of 8 consecutive pulses delivered at 1 Hz. (**C-D**) Correlation between change in mABP (**C**) or SpA BF (**D**) and TNF-α AUC evoked by stimulation at relevant IPG output. Data in C and D are individual replicates.

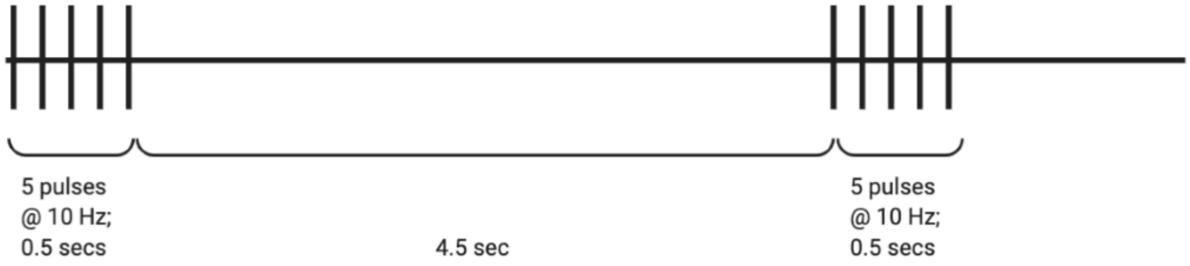

Figure S13. Graphical Representation of the 10 Hz Burst Paradigm. Stimulation is applied at 10 Hz with a 0.5 s microburst active period and a 5.0 s total microburst period, i.e. stimulation is applied for 0.5 s at 10 Hz (i.e. 5 pulses) and then there is an interval of 4.5 s before stimulation is repeated.

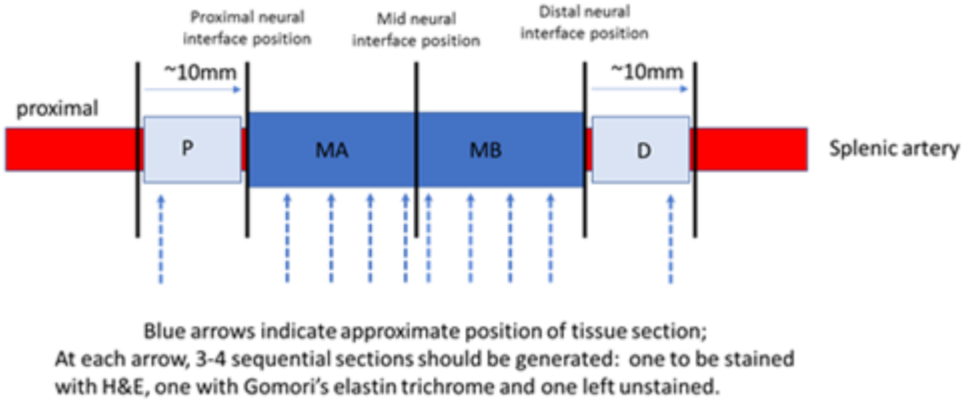

Figure S14. **Diagram of splenic artery processing for histology.** Four tissue blocks (P, MA, MB and D) are indicated.

3 Supplementary Tables

Table S1 - Naïve Phase Hematology and Biochemistry – excel file

Table S2 – Endotoxemia Phase Hematology and Biochemistry – excel file

Table S3 – SPMs in chronic pigs – separate file

Table S4 – SPMs in acute pigs – separate file

**Amplitude Range (mA)**	0 to 40 mA
**Pulse Width (µs)**	**Acute terminal eCAP & NA:**<br>400-1000 µs<br>**Chronic:**<br>980-1980 µs
**Total Charge (µC)**<br>**(mA*µs)**	**Acute terminal eCAP, NA and SPMs:**<br>Up to 40 µC<br>**Chronic:**<br>15 µC (15.3 mA; 980 µs)<br>40 µC (20 mA; 1980 µs)
**Waveform:**	Bipolar, Biphasic, Symmetrical
**Frequency (Hz)**	**Acute terminal eCAP:**<br>1, 10 or 30 Hz continuous or 10 Hz burst<br>**Acute terminal NA:**<br>10 Hz continuous or 10 Hz burst<br>**Chronic:**<br>10 Hz continuous or 10 Hz burst
**Paradigm**	**Continuous:**<br>Continuous stimulation<br>**Burst:**<br>Microburst active period = 0.5 s<br>Microburst period = 0.5 s<br>i.e. 0.5 s on; 4.5 s off

Table S5. Stimulation Parameters used across the studies: in the acute terminal and chronic conscious studies.

Antibody	Bio-Rad Catalogue no.	Conjugated probe	Isotype	Dilution
Mouse IgG1 negative control	MCA928F	FITC	IgG1	1:10
Mouse IgG1 negative control	MCA928PE	PE	IgG1	1:10
Mouse IgG2b negative control	MCA691F	FITC	IgG2b	1:10
Mouse IgG2a negative control	MCA929PE	PE	IgG2a	1:10
CD4	MCA6045F	FITC	IgG1	1:10
wCD8 Alpha	MCA1223PE	PE	IgG2a	1:10
CD14	MCA1218F	FITC	IgG2b	1:10
CD16	MCA1971PE	PE	IgG1	1:10
CD172a	MCA2312F	FITC	IgG1	1:10
CD163	MCA2311PE	PE	IgG1	1:10

Table S6. Antibody panels used for Flow Cytometry.

Grade	Descriptor	Nerve fascicles	Connective tissue
1	Minimal	Denotes a low-level change, affecting less than 5% of the tissue.	≤25% of the area of interest is affected by the change.
2	Mild	Denotes a lesion with 5 to 15% of the tissue affected.	25%-50% of the area of interest is affected by the change.
3	Moderate	Denotes a 15 to 50% of the tissue affected.	50%-75% of the area of interest is affected by the change.
4	Marked	Denotes a pronounced microscopic change with approximately 50 to 75% of the tissue affected.	75%-100% of the area of interest is affected by the change.
5	Severe	Denotes a very pronounced microscopic change with greater than 75% of the tissue affected.	

Table S7. Histopathology Grading Scheme for Tissue Sections of Splenic Artery.
