# Supplementary material for: Splenic Nerve Neuromodulation Reduces Inflammation and Promotes Resolution in Chronically Implanted Pigs
Source: Front Immunol. 2021 Mar 29;12:649786. doi: 10.3389/fimmu.2021.649786 (PMC8043071; doi:10.3389/fimmu.2021.649786)
Supplement: Supplementary file 4 [file Table_3.docx]

|  |  |  | **Plasma lipid mediators concentration (pg/mL)** | | | | | | | | | | | | | | | | | | | | | | | |
| --- | --- | --- | --- | --- | --- | --- | --- | --- | --- | --- | --- | --- | --- | --- | --- | --- | --- | --- | --- | --- | --- | --- | --- | --- | --- | --- |
|  |  |  | **Sham** | | | | | | | | | | | | **Stimulated** | | | | | | | | | | | |
| **DHA bioactive metabolome** | **Q1** | **Q3** | **0h** | | | **0.5h** | | | **3h** | | | **24h** | | | **0h** | | | **0.5h** | | | **3h** | | | **24h** | | |
| RvD1 | 375 | 215 | 1.05 | ± | 0.73 | 2.56 | ± | 1.89 | 0.00 | ± | 0.00 | 2.94 | ± | 2.27 | 1.98 | ± | 1.82 | 1.16 | ± | 0.82 | 7.68 | ± | 3.61 | 6.21 | ± | 3.41 |
| RvD2 | 375 | 215 | 0.66 | ± | 0.73 | 1.21 | ± | 0.62 | 0.44 | ± | 0.48 | 0.59 | ± | 0.66 | 0.91 | ± | 1.02 | 1.31 | ± | 0.91 | 1.01 | ± | 1.12 | 1.37 | ± | 0.77 |
| RvD3 | 375 | 147 |  | - |  |  | - |  |  | - |  | 0.09 | ± | 0.10 |  | - |  |  | - |  |  | - |  |  | - |  |
| RvD4 | 375 | 225 | 79.07 | ± | 39.42 | 208.06 | ± | 187.02 | 266.98 | ± | 279.84 | 57.60 | ± | 26.89 | 44.79 | ± | 24.34 | 56.07 | ± | 28.15 | 148.08 | ± | 125.47 | 80.19 | ± | 68.53 |
| RvD5 | 359 | 199 | 0.93 | ± | 0.37 | 1.06 | ± | 0.30 | 0.55 | ± | 0.28 | 1.91 | ± | 1.16 | 1.28 | ± | 0.56 | 0.91 | ± | 0.48 | 0.81 | ± | 0.57 | 1.15 | ± | 0.95 |
| RvD6 | 359 | 159 | 1.14 | ± | 0.34 | 1.32 | ± | 0.40 | 0.89 | ± | 0.24 | 1.20 | ± | 0.62 | 1.38 | ± | 0.85 | 2.07 | ± | 0.96 | 1.08 | ± | 0.94 | 1.23 | ± | 0.83 |
| 17R-RvD1 | 375 | 215 |  | - |  |  | - |  |  | - |  |  | - |  |  | - |  |  | - |  |  | - |  |  | - |  |
| 17R-RvD3 | 375 | 147 |  | - |  |  | - |  |  | - |  |  | - |  |  | - |  |  | - |  |  | - |  |  | - |  |
|  |  |  |  |  |  |  |  |  |  |  |  |  |  |  |  |  |  |  |  |  |  |  |  |  |  |  |
| PD1 | 359 | 153 | 20.20 | ± | 20.48 | 1.26 | ± | 0.66 | 1.20 | ± | 0.49 | 0.52 | ± | 0.40 | 2.03 | ± | 1.08 | 1.28 | ± | 1.10 | 13.39 | ± | 14.65 | 1.45 | ± | 0.93 |
| 17R-PD1 | 359 | 137 | 0.34 | ± | 0.37 |  | - |  |  | - |  |  | - |  | 0.13 | ± | 0.14 |  | - |  |  | - |  |  | - |  |
| 10S,17S-diHDHA | 359 | 153 | 1.49 | ± | 0.87 | 1.52 | ± | 0.54 | 0.88 | ± | 0.70 | 2.59 | ± | 1.14 | 1.06 | ± | 0.74 | 1.86 | ± | 1.30 | 0.17 | ± | 0.19 | 3.61 | ± | 1.88 |
| 22-OH-PD1 | 375 | 153 | 7.40 | ± | 7.49 | 5.28 | ± | 4.19 | 0.46 | ± | 0.35 | 1.16 | ± | 1.29 | 1.46 | ± | 0.62 | 0.58 | ± | 0.41 | 0.39 | ± | 0.43 |  | - |  |
|  |  |  |  |  |  |  |  |  |  |  |  |  |  |  |  |  |  |  |  |  |  |  |  |  |  |  |
| PCTR1 | 650 | 231 |  | - |  |  | - |  |  | - |  |  | - |  |  | - |  |  | - |  |  | - |  |  | - |  |
| PCTR2 | 521 | 231 |  | - |  |  | - |  |  | - |  |  | - |  |  | - |  |  | - |  |  | - |  |  | - |  |
| PCTR3 | 464 | 231 |  | - |  |  | - |  |  | - |  |  | - |  |  | - |  |  | - |  |  | - |  |  | - |  |
|  |  |  |  |  |  |  |  |  |  |  |  |  |  |  |  |  |  |  |  |  |  |  |  |  |  |  |
| MaR1 | 359 | 177 | 0.63 | ± | 0.45 | 0.57 | ± | 0.63 | 0.71 | ± | 0.50 | 0.82 | ± | 0.59 | 0.71 | ± | 0.62 | 0.17 | ± | 0.19 | 0.88 | ± | 0.60 | 0.96 | ± | 0.74 |
| MaR2 | 359 | 191 |  | - |  | 1.10 | ± | 0.78 | 1.45 | ± | 1.01 |  | - |  |  | - |  | 1.35 | ± | 0.93 | 0.40 | ± | 0.45 |  | - |  |
| 22-OH-MaR1 | 375 | 221 | 10.52 | ± | 7.41 | 2.55 | ± | 1.84 | 6.15 | ± | 3.23 | 7.30 | ± | 4.42 | 7.32 | ± | 4.13 | 3.72 | ± | 2.05 | 2.16 | ± | 1.49 | 8.72 | ± | 5.61 |
| 14-oxo-MaR1 | 357 | 248 |  | - |  |  | - |  |  | - |  |  | - |  |  | - |  |  | - |  |  | - |  |  | - |  |
| 7S,14S-diHDHA | 359 | 221 | 0.25 | ± | 0.27 | 0.64 | ± | 0.46 | 0.73 | ± | 0.37 |  | - |  |  | - |  | 0.96 | ± | 0.79 | 0.79 | ± | 0.88 | 0.83 | ± | 0.61 |
| 4S,14S-diHDHA | 359 | 159 |  | - |  | 0.30 | ± | 0.33 |  | - |  | 0.32 | ± | 0.35 |  | - |  | 0.43 | ± | 0.48 |  | - |  | 0.33 | ± | 0.37 |
|  |  |  |  |  |  |  |  |  |  |  |  |  |  |  |  |  |  |  |  |  |  |  |  |  |  |  |
| MCTR1 | 650 | 191 |  | - |  |  | - |  |  | - |  |  | - |  |  | - |  |  | - |  |  | - |  |  | - |  |
| MCTR2 | 521 | 191 |  | - |  |  | - |  |  | - |  |  | - |  |  | - |  |  | - |  |  | - |  |  | - |  |
| MCTR3 | 464 | 191 | 9.20 | ± | 2.82 | 3.14 | ± | 0.81 | 3.20 | ± | 0.93 | 3.84 | ± | 1.89 | 4.06 | ± | 1.48 | 4.44 | ± | 2.12 | 3.42 | ± | 1.63 | 4.09 | ± | 2.57 |
| **n-3 DPA bioactive metabolome** |  |  |  |  |  |  |  |  |  |  |  |  |  |  |  |  |  |  |  |  |  |  |  |  |  |  |
| RvT1 | 377 | 211 | 2.20 | ± | 1.77 | 1.80 | ± | 1.98 | 3.21 | ± | 2.22 | 5.75 | ± | 1.83 | 0.82 | ± | 0.91 |  | - |  | 2.63 | ± | 1.80 | 1.94 | ± | 2.17 |
| RvT2 | 377 | 197 |  | - |  |  | - |  |  | - |  |  | - |  |  | - |  |  | - |  |  | - |  |  | - |  |
| RvT3 | 377 | 197 |  | - |  |  | - |  |  | - |  |  | - |  |  | - |  |  | - |  |  | - |  |  | - |  |
| RvT4 | 359 | 211 | 0.72 | ± | 0.37 | 0.61 | ± | 0.34 | 1.42 | ± | 0.31 | 1.14 | ± | 0.35 | 1.28 | ± | 0.14 | 0.98 | ± | 0.31 | 0.80 | ± | 0.39 | 1.61 | ± | 0.50 |
|  |  |  |  |  |  |  |  |  |  |  |  |  |  |  |  |  |  |  |  |  |  |  |  |  |  |  |
| RvD1_n-3 DPA_ | 377 | 215 |  | - |  |  | - |  |  | - |  |  | - |  |  | - |  |  | - |  |  | - |  |  | - |  |
| RvD2_n-3 DPA_ | 377 | 233 |  | - |  |  | - |  |  | - |  |  | - |  |  | - |  |  | - |  |  | - |  |  | - |  |
| RvD5_n-3 DPA_ | 361 | 199 | 1.42 | ± | 0.74 | 1.66 | ± | 0.89 | 0.52 | ± | 0.57 | 0.43 | ± | 0.48 | 0.32 | ± | 0.36 | 0.89 | ± | 0.65 | 0.29 | ± | 0.32 | 1.77 | ± | 0.59 |
|  |  |  |  |  |  |  |  |  |  |  |  |  |  |  |  |  |  |  |  |  |  |  |  |  |  |  |
| PD1_n-3 DPA_ | 361 | 183 | 0.45 | ± | 0.23 | 0.95 | ± | 0.28 | 0.48 | ± | 0.25 | 0.34 | ± | 0.25 | 0.13 | ± | 0.15 |  | - |  | 0.17 | ± | 0.19 | 0.10 | ± | 0.11 |
| 10S,17S-diHDPA | 361 | 183 | 4.24 | ± | 0.57 | 4.39 | ± | 0.53 | 4.57 | ± | 0.57 | 4.81 | ± | 0.72 | 4.26 | ± | 0.89 | 4.80 | ± | 0.57 | 4.62 | ± | 0.73 | 4.85 | ± | 0.55 |
|  |  |  |  |  |  |  |  |  |  |  |  |  |  |  |  |  |  |  |  |  |  |  |  |  |  |  |
| MaR1_n-3 DPA_ | 361 | 223 | 0.42 | ± | 0.46 | 0.18 | ± | 0.20 | 0.81 | ± | 0.41 | 0.28 | ± | 0.31 | 1.27 | ± | 0.70 | 1.29 | ± | 0.54 | 1.00 | ± | 0.74 | 0.20 | ± | 0.22 |
| 7S,14S-diHDPA | 361 | 223 |  | - |  |  | - |  |  | - |  |  | - |  |  | - |  |  | - |  |  | - |  |  | - |  |
| **EPA bioactive metabolome** |  |  |  |  |  |  |  |  |  |  |  |  |  |  |  |  |  |  |  |  |  |  |  |  |  |  |
| RvE1 | 349 | 161 | 7.02 | ± | 1.60 | 7.75 | ± | 2.43 | 6.99 | ± | 3.75 | 5.28 | ± | 0.83 | 8.32 | ± | 2.52 | 7.72 | ± | 2.72 | 7.12 | ± | 1.93 | 8.25 | ± | 1.09 |
| RvE2 | 333 | 159 | 0.38 | ± | 0.41 | 0.32 | ± | 0.35 | 0.42 | ± | 0.46 |  | - |  | 0.30 | ± | 0.33 | 0.55 | ± | 0.62 | 0.79 | ± | 0.38 | 0.43 | ± | 0.48 |
| RvE3 | 333 | 201 | 1.94 | ± | 0.99 | 3.25 | ± | 1.39 | 3.01 | ± | 1.38 | 1.41 | ± | 0.71 | 2.51 | ± | 1.25 | 4.72 | ± | 1.52 | 3.55 | ± | 1.43 | 5.26 | ± | 1.40 |
| **AA bioactive metabolome** |  |  |  |  |  |  |  |  |  |  |  |  |  |  |  |  |  |  |  |  |  |  |  |  |  |  |
| LXA_4_ | 351 | 115 |  | - |  |  | - |  |  | - |  |  | - |  |  | - |  |  | - |  |  | - |  |  | - |  |
| LXB_4_ | 351 | 115 | 84.42 | ± | 57.92 | 26.58 | ± | 9.88 | 111.42 | ± | 57.12 | 97.75 | ± | 80.45 | 72.57 | ± | 29.59 | 68.15 | ± | 24.00 | 214.91 | ± | 174.81 | 143.19 | ± | 72.07 |
| 5S,15S-diHETE | 335 | 115 |  | - |  | 2.67 | ± | 1.90 | 7.86 | ± | 4.50 | 6.03 | ± | 2.89 |  | - |  | 2.72 | ± | 1.87 | 5.54 | ± | 3.32 | 2.90 | ± | 3.24 |
| 15epi-LXA_4_ | 351 | 217 | 3.95 | ± | 3.45 | 4.57 | ± | 2.57 | 6.82 | ± | 3.58 | 6.91 | ± | 5.81 | 0.27 | ± | 0.30 | 4.69 | ± | 3.27 | 15.73 | ± | 10.92 | 7.06 | ± | 6.68 |
| 15epi-LXB_4_ | 351 | 221 |  | - |  |  | - |  |  | - |  |  | - |  |  | - |  |  | - |  |  | - |  |  | - |  |
| 13,14-dehydro, 15-oxo-LXA_4_ | 351 | 217 |  | - |  |  | - |  |  | - |  |  | - |  |  | - |  |  | - |  |  | - |  |  | - |  |
| 15-oxo-LXA_4_ | 349 | 115 |  | - |  |  | - |  |  | - |  |  | - |  |  | - |  |  | - |  |  | - |  |  | - |  |
|  |  |  |  |  |  |  |  |  |  |  |  |  |  |  |  |  |  |  |  |  |  |  |  |  |  |  |
| LTB_4_ | 335 | 195 | 10.98 | ± | 1.78 | 8.73 | ± | 1.23 | 8.36 | ± | 1.20 | 9.85 | ± | 0.92 | 10.98 | ± | 2.66 | 11.09 | ± | 2.96 | 9.89 | ± | 2.99 | 10.84 | ± | 1.90 |
| 5S,12S-diHETE | 335 | 195 |  | - |  |  | - |  |  | - |  |  | - |  |  | - |  |  | - |  |  | - |  |  | - |  |
| 6-trans-LTB_4_ | 335 | 195 |  | - |  | 0.54 | ± | 0.32 | 0.63 | ± | 0.32 | 0.13 | ± | 0.15 | 0.33 | ± | 0.37 | 0.33 | ± | 0.17 | 0.64 | ± | 0.31 | 0.41 | ± | 0.20 |
| 6-trans,12-epi-LTB_4_ | 335 | 195 |  | - |  | 0.22 | ± | 0.24 |  | - |  | 0.50 | ± | 0.25 | 0.17 | ± | 0.19 | 0.07 | ± | 0.08 |  | - |  | 0.17 | ± | 0.19 |
| 20-OH-LTB_4_ | 351 | 195 | 0.66 | ± | 0.47 | 0.33 | ± | 0.37 |  | - |  |  | - |  |  | - |  | 0.70 | ± | 0.53 | 1.17 | ± | 0.89 | 0.24 | ± | 0.27 |
|  |  |  |  |  |  |  |  |  |  |  |  |  |  |  |  |  |  |  |  |  |  |  |  |  |  |  |
| LTC_4_ | 626 | 189 |  | - |  |  | - |  |  | - |  |  | - |  |  | - |  |  | - |  |  | - |  |  | - |  |
| LTD_4_ | 497 | 189 |  | - |  |  | - |  |  | - |  |  | - |  |  | - |  |  | - |  |  | - |  |  | - |  |
| LTE_4_ | 440 | 189 | 6.97 | ± | 1.98 | 5.96 | ± | 1.17 | 4.64 | ± | 0.96 | 5.63 | ± | 1.34 | 4.30 | ± | 1.16 | 3.62 | ± | 0.43 | 3.94 | ± | 1.33 | 5.33 | ± | 1.45 |
|  |  |  |  |  |  |  |  |  |  |  |  |  |  |  |  |  |  |  |  |  |  |  |  |  |  |  |
| PGD_2_ | 351 | 189 | 12.85 | ± | 2.58 | 15.31 | ± | 5.29 | 16.54 | ± | 5.44 | 9.81 | ± | 3.99 | 6.53 | ± | 1.87 | 8.77 | ± | 1.80 | 12.50 | ± | 2.32 | 6.84 | ± | 1.72 |
| PGE_2_ | 351 | 189 | 7.08 | ± | 1.61 | 7.41 | ± | 1.04 | 9.99 | ± | 3.79 | 10.35 | ± | 6.35 | 2.86 | ± | 0.65 | 4.46 | ± | 0.83 | 5.97 | ± | 1.82 | 5.62 | ± | 1.12 |
| PGF_2α_ | 353 | 193 | 40.15 | ± | 5.06 | 29.26 | ± | 4.29 | 31.95 | ± | 7.05 | 29.58 | ± | 10.22 | 32.69 | ± | 7.12 | 30.82 | ± | 6.83 | 33.88 | ± | 10.56 | 40.51 | ± | 10.00 |
| TxB_2_ | 369 | 169 | 68.38 | ± | 23.06 | 157.25 | ± | 16.05 | 78.15 | ± | 9.37 | 49.50 | ± | 8.27 | 37.84 | ± | 7.62 | 104.49 | ± | 17.78 | 73.78 | ± | 14.80 | 40.63 | ± | 7.38 |

**Table S3:** **Quantification of Plasma Lipid Mediators in Conscious Pigs Following Chronic SpN Neuromodulation.** Porcine SpNs were stimulated chronically prior LPS challenge. Plasma was collected at various intervals post LPS challenge and LM were investigated using LC-MS/MS-based profiling. Results are expressed as pg/mL, mean±sem. n=5 for Sham group and n=6 for Stimulated group. -, below limit, Q1, parent ion, Q3, daughter ion.

|  |  |  | **Plasma lipid mediators concentration (pg/mL)** | | | | | | | | | | | | | | | | | |
| --- | --- | --- | --- | --- | --- | --- | --- | --- | --- | --- | --- | --- | --- | --- | --- | --- | --- | --- | --- | --- |
|  |  |  | **Sham** | | | | | | | | | **Stimulated** | | | | | | | | |
| **DHA bioactive metabolome** | **Q1** | **Q3** | **0min** | | | **30min** | | | **120min** | | | **0min** | | | **30min** | | | **120min** | | |
| RvD1 | 375 | 233 | 0.24 | ± | 0.18 | 0.13 | ± | 0.15 |  | - |  |  | - |  | 0.12 | ± | 0.13 | 0.16 | ± | 0.18 |
| RvD2 | 375 | 215 | 0.62 | ± | 0.50 | 0.56 | ± | 0.50 | 0.20 | ± | 0.21 |  | - |  | 0.39 | ± | 0.27 | 0.21 | ± | 0.23 |
| RvD3 | 375 | 147 | 0.03 | ± | 0.04 | 0.00 | ± | 0.00 | 0.31 | ± | 0.34 | 0.21 | ± | 0.10 |  | - |  | 0.07 | ± | 0.05 |
| RvD4 | 375 | 101 | 0.69 | ± | 0.34 | 0.75 | ± | 0.38 | 0.80 | ± | 0.29 | 0.92 | ± | 0.42 | 0.61 | ± | 0.27 | 0.56 | ± | 0.30 |
| RvD5 | 359 | 199 | 0.19 | ± | 0.15 | 0.82 | ± | 0.30 | 0.55 | ± | 0.30 | 0.21 | ± | 0.11 | 0.53 | ± | 0.26 | 0.34 | ± | 0.24 |
| RvD6 | 359 | 159 | 2.14 | ± | 0.53 | 1.73 | ± | 0.64 | 1.67 | ± | 0.58 | 1.93 | ± | 0.23 | 1.66 | ± | 0.41 | 1.25 | ± | 0.45 |
| 17R-RvD1 | 375 | 215 | 0.02 | ± | 0.03 |  | - |  | 0.18 | ± | 0.15 | 0.12 | ± | 0.14 | 0.30 | ± | 0.17 | 0.24 | ± | 0.13 |
| 17R-RvD3 | 375 | 137 |  | - |  | 0.45 | ± | 0.49 | 0.05 | ± | 0.05 |  | - |  |  | - |  |  | - |  |
|  |  |  |  |  |  |  |  |  |  |  |  |  |  |  |  |  |  |  |  |  |
| PD1 | 359 | 153 | 0.36 | ± | 0.25 | 0.72 | ± | 0.33 | 0.69 | ± | 0.27 | 0.48 | ± | 0.21 | 0.15 | ± | 0.16 | 0.16 | ± | 0.17 |
| 17R-PD1 | 359 | 153 |  | - |  |  | - |  |  | - |  |  | - |  | 0.19 | ± | 0.21 | 0.05 | ± | 0.06 |
| 10S,17S-diHDHA | 359 | 153 | 0.16 | ± | 0.13 | 0.16 | ± | 0.18 | 0.45 | ± | 0.25 | 0.17 | ± | 0.12 | 0.86 | ± | 0.25 | 0.60 | ± | 0.17 |
| 22-OH-PD1 | 375 | 153 |  | - |  |  | - |  |  | - |  |  | - |  | 0.09 | ± | 0.06 | 0.06 | ± | 0.07 |
|  |  |  |  |  |  |  |  |  |  |  |  |  |  |  |  |  |  |  |  |  |
| PCTR1 | 650 | 231 |  | - |  |  | - |  |  | - |  |  | - |  |  | - |  |  | - |  |
| PCTR2 | 521 | 231 |  | - |  |  | - |  |  | - |  |  | - |  |  | - |  |  | - |  |
| PCTR3 | 464 | 231 | 0.60 | ± | 0.32 | 0.39 | ± | 0.29 | 0.97 | ± | 0.48 | 0.66 | ± | 0.46 | 1.26 | ± | 0.58 | 0.95 | ± | 0.48 |
|  |  |  |  |  |  |  |  |  |  |  |  |  |  |  |  |  |  |  |  |  |
| MaR1 | 359 | 177 | 1.11 | ± | 0.93 | 2.50 | ± | 1.34 | 4.25 | ± | 2.34 | 0.51 | ± | 0.56 | 3.06 | ± | 0.75 | 1.35 | ± | 0.69 |
| MaR2 | 359 | 191 |  | - |  | 0.41 | ± | 0.44 |  | - |  |  | - |  |  | - |  |  | - |  |
| 22-OH-MaR1 | 375 | 221 |  | - |  |  | - |  |  | - |  |  | - |  |  | - |  |  | - |  |
| 14-oxo-MaR1 | 357 | 248 |  | - |  |  | - |  |  | - |  |  | - |  |  | - |  |  | - |  |
| 7S,14S-diHDHA | 359 | 221 | 1.58 | ± | 1.51 | 5.05 | ± | 3.20 |  | - |  | 3.20 | ± | 1.36 | 2.68 | ± | 1.32 | 2.96 | ± | 1.16 |
| 4S,14S-diHDHA | 359 | 101 | 0.25 | ± | 0.20 | 0.52 | ± | 0.31 | 0.29 | ± | 0.21 | 0.12 | ± | 0.13 | 0.17 | ± | 0.18 | 0.33 | ± | 0.23 |
|  |  |  |  |  |  |  |  |  |  |  |  |  |  |  |  |  |  |  |  |  |
| MCTR1 | 650 | 191 |  | - |  |  | - |  |  | - |  |  | - |  |  | - |  |  | - |  |
| MCTR2 | 521 | 191 |  | - |  |  | - |  |  | - |  |  | - |  |  | - |  |  | - |  |
| MCTR3 | 464 | 191 | 1.90 | ± | 0.92 | 2.32 | ± | 0.63 | 3.02 | ± | 1.60 | 2.48 | ± | 0.46 | 3.66 | ± | 0.64 | 2.95 | ± | 0.60 |
| **n-3 DPA bioactive metabolome** |  |  |  |  |  |  |  |  |  |  |  |  |  |  |  |  |  |  |  |  |
| RvT1 | 377 | 211 | 1.97 | ± | 2.16 | 3.75 | ± | 3.03 | 0.37 | ± | 0.41 | 1.42 | ± | 0.88 |  | - |  |  | - |  |
| RvT2 | 377 | 197 |  | - |  |  | - |  |  | - |  | 0.71 | ± | 0.77 |  | - |  |  | - |  |
| RvT3 | 377 | 173 |  | - |  |  | - |  |  | - |  |  | - |  |  | - |  |  | - |  |
| RvT4 | 359 | 211 |  | - |  |  | - |  |  | - |  |  | - |  | 0.21 | ± | 0.23 |  | - |  |
|  |  |  |  |  |  |  |  |  |  |  |  |  |  |  |  |  |  |  |  |  |
| RvD1_n-3 DPA_ | 377 | 143 | 0.09 | ± | 0.06 | 0.24 | ± | 0.26 |  | - |  | 0.07 | ± | 0.08 | 0.24 | ± | 0.13 | 0.15 | ± | 0.16 |
| RvD2_n-3 DPA_ | 377 | 261 | 0.38 | ± | 0.21 | 1.39 | ± | 0.58 | 1.05 | ± | 0.54 | 0.15 | ± | 0.17 | 1.13 | ± | 0.53 | 1.25 | ± | 0.67 |
| RvD5_n-3 DPA_ | 361 | 199 | 1.55 | ± | 1.17 | 0.46 | ± | 0.32 | 0.95 | ± | 1.04 | 0.40 | ± | 0.28 | 1.32 | ± | 0.74 |  | - |  |
|  |  |  |  |  |  |  |  |  |  |  |  |  |  |  |  |  |  |  |  |  |
| PD1_n-3 DPA_ | 361 | 183 | 0.11 | ± | 0.12 | 0.09 | ± | 0.10 | 0.06 | ± | 0.06 | 0.06 | ± | 0.07 |  | - |  |  | - |  |
| 10S,17S-diHDPA | 361 | 155 | 0.34 | ± | 0.18 | 0.95 | ± | 0.34 | 0.22 | ± | 0.15 | 0.76 | ± | 0.22 | 0.33 | ± | 0.23 | 0.10 | ± | 0.11 |
|  |  |  |  |  |  |  |  |  |  |  |  |  |  |  |  |  |  |  |  |  |
| MaR1_n-3 DPA_ | 361 | 223 | 0.15 | ± | 0.16 | 0.12 | ± | 0.13 | 0.43 | ± | 0.31 | 0.16 | ± | 0.18 | 0.35 | ± | 0.26 | 0.24 | ± | 0.26 |
| 7S,14S-diHDPA | 361 | 223 | 0.35 | ± | 0.26 |  | - |  | 0.14 | ± | 0.16 |  | - |  |  | - |  |  | - |  |
| **EPA bioactive metabolome** |  |  |  |  |  |  |  |  |  |  |  |  |  |  |  |  |  |  |  |  |
| RvE1 | 349 | 161 |  | - |  |  | - |  |  | - |  |  | - |  |  | - |  |  | - |  |
| RvE2 | 333 | 159 | 0.12 | ± | 0.14 | 0.35 | ± | 0.25 |  | - |  |  | - |  | 0.18 | ± | 0.20 |  | - |  |
| RvE3 | 333 | 275 | 8.09 | ± | 2.99 | 8.46 | ± | 2.98 | 5.87 | ± | 3.19 | 6.81 | ± | 1.09 | 4.34 | ± | 1.69 | 3.82 | ± | 1.44 |
| **AA bioactive metabolome** |  |  |  |  |  |  |  |  |  |  |  |  |  |  |  |  |  |  |  |  |
| LXA_4_ | 351 | 217 | 0.04 | ± | 0.05 |  | - |  |  | - |  |  | - |  |  | - |  |  | - |  |
| LXB_4_ | 351 | 115 | 10.69 | ± | 3.28 | 14.71 | ± | 7.67 | 22.79 | ± | 11.50 | 8.77 | ± | 1.43 | 7.06 | ± | 0.80 | 9.72 | ± | 5.73 |
| 5S,15S-diHETE | 335 | 235 | 12.27 | ± | 3.46 | 11.71 | ± | 5.31 | 8.18 | ± | 3.27 | 13.02 | ± | 2.71 | 9.89 | ± | 1.72 | 11.54 | ± | 4.08 |
| 15epi-LXA_4_ | 351 | 217 | 4.75 | ± | 1.47 | 12.27 | ± | 7.52 | 7.58 | ± | 5.65 | 3.19 | ± | 0.35 | 17.29 | ± | 15.61 | 5.66 | ± | 4.68 |
| 15epi-LXB_4_ | 351 | 221 | 1.09 | ± | 0.48 | 0.25 | ± | 0.18 | 0.18 | ± | 0.19 | 0.74 | ± | 0.27 | 1.89 | ± | 1.31 | 0.59 | ± | 0.29 |
| 13,14-dehydro, 15-oxo-LXA_4_ | 351 | 217 | 0.32 | ± | 0.31 | 1.24 | ± | 1.29 | 0.96 | ± | 0.78 | 0.44 | ± | 0.25 |  | - |  | 0.18 | ± | 0.06 |
| 15-oxo-LXA_4_ | 349 | 115 | 0.16 | ± | 0.09 | 0.07 | ± | 0.08 | 0.22 | ± | 0.17 | 0.28 | ± | 0.12 | 0.33 | ± | 0.15 | 0.25 | ± | 0.15 |
|  |  |  |  |  |  |  |  |  |  |  |  |  |  |  |  | - |  |  | - |  |
| LTB_4_ | 335 | 195 | 20.26 | ± | 5.33 | 23.14 | ± | 6.61 | 15.65 | ± | 3.67 | 15.36 | ± | 4.55 | 16.16 | ± | 3.22 | 10.96 | ± | 2.80 |
| 5S,12S-diHETE | 335 | 195 | 0.74 | ± | 0.81 | 2.19 | ± | 1.73 |  | - |  | 0.41 | ± | 0.45 |  | - |  | 0.80 | ± | 0.68 |
| 6-trans-LTB_4_ | 335 | 195 | 1.11 | ± | 0.83 | 0.79 | ± | 0.64 | 0.36 | ± | 0.30 | 0.61 | ± | 0.30 | 1.02 | ± | 0.32 | 0.18 | ± | 0.13 |
| 6-trans,12-epi-LTB_4_ | 335 | 195 |  | - |  |  | - |  |  | - |  |  | - |  |  | - |  |  | - |  |
| 20-OH-LTB_4_ | 351 | 195 | 0.09 | ± | 0.07 | 0.06 | ± | 0.07 | 0.25 | ± | 0.13 | 0.12 | ± | 0.06 | 0.18 | ± | 0.09 | 0.04 | ± | 0.05 |
|  |  |  |  |  |  |  |  |  |  |  |  |  |  |  |  |  |  |  |  |  |
| LTC_4_ | 626 | 189 |  | - |  |  | - |  |  | - |  |  | - |  |  | - |  |  | - |  |
| LTD_4_ | 497 | 189 |  | - |  |  | - |  |  | - |  |  | - |  |  | - |  |  | - |  |
| LTE_4_ | 440 | 189 | 3.19 | ± | 0.72 | 4.18 | ± | 0.70 | 5.18 | ± | 1.08 | 4.13 | ± | 0.99 | 5.63 | ± | 1.19 | 5.97 | ± | 1.22 |
|  |  |  |  |  |  |  |  |  |  |  |  |  |  |  |  |  |  |  |  |  |
| PGD_2_ | 351 | 189 | 4.90 | ± | 1.11 | 21.73 | ± | 12.55 | 12.90 | ± | 5.52 | 3.45 | ± | 0.34 | 31.11 | ± | 21.73 | 13.45 | ± | 9.79 |
| PGE_2_ | 351 | 189 | 3.08 | ± | 1.04 | 8.07 | ± | 3.44 | 6.98 | ± | 1.97 | 2.31 | ± | 0.39 | 9.82 | ± | 5.82 | 5.54 | ± | 2.85 |
| PGF_2α_ | 353 | 193 | 13.96 | ± | 4.24 | 30.26 | ± | 6.96 | 15.71 | ± | 3.77 | 9.54 | ± | 1.86 | 31.63 | ± | 12.04 | 10.46 | ± | 1.49 |
| TxB_2_ | 369 | 169 | 0.06 | ± | 0.06 | 2.62 | ± | 1.32 | 2.26 | ± | 0.74 | 0.54 | ± | 0.38 | 9.39 | ± | 4.46 | 3.28 | ± | 1.41 |

**Table S4**: **Quantification of Plasma Lipid Mediators in Anaesthetised Pigs Following Acute SpN Neuromodulation.** Porcine SpNs were stimulated once prior LPS challenge. Plasma was collected at various intervals post LPS challenge and LM were investigated using LC-MS/MS-based profiling. Results are expressed as pg/mL, mean±sem. n=6 for Sham group and n=6 for Stimulated group. -, below limit, Q1, parent ion, Q3, daughter ion.
